# Novel Insect Leaf-Mining after the End-Cretaceous Extinction and the Demise of Cretaceous Leaf Miners, Great Plains, USA

**DOI:** 10.1371/journal.pone.0103542

**Published:** 2014-07-24

**Authors:** Michael P. Donovan, Peter Wilf, Conrad C. Labandeira, Kirk R. Johnson, Daniel J. Peppe

**Affiliations:** 1 Department of Geosciences, Pennsylvania State University, University Park, Pennsylvania, United States of America; 2 Department of Paleobiology, National Museum of Natural History, Smithsonian Institution, Washington, District of Columbia, United States of America; 3 Department of Entomology and BEES Program, University of Maryland, College Park, Maryland, United States of America; 4 National Museum of Natural History, Smithsonian Institution, Washington, District of Columbia, United States of America; 5 Department of Geology, Baylor University, Waco, Texas, United States of America; Raymond M. Alf Museum of Paleontology, United States of America

## Abstract

Plant and associated insect-damage diversity in the western U.S.A. decreased significantly at the Cretaceous-Paleogene (K-Pg) boundary and remained low until the late Paleocene. However, the Mexican Hat locality (ca. 65 Ma) in southeastern Montana, with a typical, low-diversity flora, uniquely exhibits high damage diversity on nearly all its host plants, when compared to all known local and regional early Paleocene sites. The same plant species show minimal damage elsewhere during the early Paleocene. We asked whether the high insect damage diversity at Mexican Hat was more likely related to the survival of Cretaceous insects from refugia or to an influx of novel Paleocene taxa. We compared damage on 1073 leaf fossils from Mexican Hat to over 9000 terminal Cretaceous leaf fossils from the Hell Creek Formation of nearby southwestern North Dakota and to over 9000 Paleocene leaf fossils from the Fort Union Formation in North Dakota, Montana, and Wyoming. We described the entire insect-feeding ichnofauna at Mexican Hat and focused our analysis on leaf mines because they are typically host-specialized and preserve a number of diagnostic morphological characters. Nine mine damage types attributable to three of the four orders of leaf-mining insects are found at Mexican Hat, six of them so far unique to the site. We found no evidence linking any of the diverse Hell Creek mines with those found at Mexican Hat, nor for the survival of any Cretaceous leaf miners over the K-Pg boundary regionally, even on well-sampled, surviving plant families. Overall, our results strongly relate the high damage diversity on the depauperate Mexican Hat flora to an influx of novel insect herbivores during the early Paleocene, possibly caused by a transient warming event and range expansion, and indicate drastic extinction rather than survivorship of Cretaceous insect taxa from refugia.

## Introduction

One of the largest and most sudden mass extinctions in Earth history occurred at the end of the Cretaceous period (66.0 Ma), triggered by an asteroid impact at Chicxulub, Mexico [1]–[5]. The paleobotanical record from the Western Interior United States shows a ca. 30% (pollen) to ca. 57% (southwestern North Dakota macrofossils) reduction in diversity at the Cretaceous-Paleogene (K-Pg) boundary [6]–[9]. The richness of insect-feeding damage on fossil leaves also decreased ca. 42% across the K-Pg boundary in North Dakota [10], [11]. Mines and galls, typically representing host-specific interactions, were disproportionally affected in the extinction [10], [11].

Paleocene floras of the Western Interior U.S.A. typically had low diversity and were dominated by widespread species [12]–[14]. For ca. 10 million years following the K-Pg extinction, floral diversity was depressed compared to the latest Cretaceous and finally began to increase during the latest Paleocene and early Eocene [15]–[17]. Insect damage diversity was also low during most of the Paleocene, but it increased to levels similar to the latest Cretaceous during late Paleocene warming, preceding the increase in plant diversity [10], [11], [16], [18]–[20].

Insect diversity generally correlates with plant diversity today [21]. Comparably, low diversity paleofloras with low diversity insect damage have been observed at all regional early Paleocene localities previously examined for insect damage, with the exception of two unusual localities: Mexican Hat (southeastern Montana) and Castle Rock (north-central Colorado) [10], [11], [19]. Castle Rock has a rainforest-like flora with high plant diversity and low insect damage diversity [19], [22], [23]. Mexican Hat has a typical, low diversity flora, but uniquely, this is paired with highly diverse insect damage across host plants [19]. Wilf et al. [19] hypothesized that these two anomalous localities represent decoupled plant and insect diversity resulting from broken food webs in the wake of a mass extinction.

Besides Mexican Hat's anomalous insect damage diversity, the locality also marks the earliest evidence for both a plant and an insect family, providing clear examples of novelty following the K-Pg extinction. The earliest well-dated evidence for Juglandaceae (walnut family) is the co-occurrence at Mexican Hat of *Polyptera manningii* fruits and *Juglandiphyllites glabra* leaflets [24], [25]. A leaf mine occurring on *Platanus raynoldsii* at Mexican Hat, *Phytomyzites biliapchaensis*, represents the earliest evidence for Agromyzidae and, by extension, the encompassing Schizophora clade of leaf-mining flies [26].

Despite Mexican Hat's singular importance for understanding the recovery of plant-insect ecosystems after mass extinction, its insect damage is only partially described [26], [27], aside from an unpublished thesis examining one plant species [27], and has not been rigorously compared to the well-preserved damage from nearby exposures of the latest Cretaceous Hell Creek Formation [19]. The purpose of this study is to determine whether the insect damage at Mexican Hat is more likely to represent Cretaceous insects from refugia or novel Paleocene taxa, and to use this information for a reassessment of the overall extinction of leaf-mining insects across the K-Pg boundary. To provide the first detailed documentation of this spectacular ichnofauna, we first describe all insect damage at Mexican Hat by host plant, and we then focus analytically on leaf mines for comparison with local and regional Cretaceous and Paleocene floras because mines commonly preserve various morphological details useful for species-equivalent comparisons. We also focus on well-sampled surviving plant families, including Platanaceae and Cercidiphyllaceae, to determine if they provided a refuge for Cretaceous insects. Links between the diverse mining fauna at Mexican Hat and local and regional Cretaceous sites currently are unknown, and determining if there are connections to the high damage diversity at Mexican Hat will allow us to reassess the regional severity of leaf miner extinction across the K-Pg boundary.

## Materials and Methods

### Ethics Statement

This study was done entirely using previously collected materials that were permanently deposited in major natural history museums, as detailed below. No new permits were required for the described study, which complied with all relevant regulations.

The Mexican Hat locality is a sombrero-shaped, isolated butte approximately 51 kilometers east of Miles City, southeastern Montana, in the Hogan Creek Quadrangle at 46.43139°N, 105.24117°W. The site is in the northeastern portion of the Powder River Basin, in the Lebo Member of the Fort Union Formation. The Lebo Member locally overlies the Tullock Member, which lies conformably above the Cretaceous Hell Creek Formation. The Lebo Member represents an early Paleocene meandering channel system that deposited sediments from channels, point bars, crevasse splays, and floodplains [28]. The early Paleocene transgression of the Cannonball Sea in the Western Interior USA may have raised base level inland, causing more flooded landscapes with increased soil water concentration and leading to deposition of mire and ponded-water facies and the fossilization of swamp and riparian forest communities [6]. The fossiliferous sediments at Mexican Hat are 2.5 m thick and are composed mostly of mudstones and thin coals, with fine sandstones at the base, all deposited in levee and floodplain environments [27], [28]. The flora most likely represents a riparian forest community adjacent to a meandering channel.

Regarding the age of the flora, there are no paleomagnetic or radiometric data from the outcrop itself; a possible volcanic ash collected there by PW did not yield useful phenocrysts (M.E. Smith, personal communication 2005). However, Belt et al. [29] reported 40 Ar/39 Ar dates of 64.0–64.7 Ma from two nearby outcrops in the Lebo Member [29]. The Mexican Hat fossil site is located approximately 15 m above the contact between the Tullock and Lebo Members (ES Belt, personal communication 2012). At Signal Butte, Montana, located 40 km west of Mexican Hat, the magnetic polarity reversal between chrons C29n and C28r occurs approximately 10 m above the Tullock-Lebo contact [30]. Because of potential differences in sedimentation rates and topography, the sediments at Mexican Hat could have been deposited either during C29n or C28r, whose reversal boundary is currently calibrated to 64.95 Ma [31]. This is consistent with the previous 40 Ar/39 Ar ages for the Lebo Member [29] in light of current calibration constants [32]. Thus, in the absence of more precise geochronologic constraints, we estimate the age of the Mexican Hat fossil assemblage to be approximately 65 Ma (1 m.y. after the K-Pg boundary).

Williams [28] collected the first fossil plant specimens from Mexican Hat in 1988 for a senior thesis study of depositional environments of Paleocene plant fossil localities in southeastern Montana. Her collection consists of ca. 400 specimens and is curated at the Yale University Peabody Museum of Natural History (YPM PB. 05250 and PB. 01790). In 1996, Lang [27] collected *Zizyphoides flabella* leaves from Mexican Hat for his doctoral thesis, which analyzed plant-insect associations in the Western Interior USA ranging from the Late Cretaceous to Eocene. From Lang's collection, the 78 specimens curated at the Denver Museum of Nature and Science were examined (DMNH, localities 1251 and 1252). Williams and Lang did not perform quantitative field censuses of the fossils at Mexican Hat, which was later done by PW and CCL, June 22 to July 2, 2004, resulting in the largest collection of Mexican Hat plant fossils. In this field census, 2219 leaves and leaflets were scored for presence or absence of insect feeding damage types (DTs), and 513 voucher specimens and 82 fragmentary or indeterminate specimens were deposited at the Smithsonian Institution National Museum of Natural History (USNM, locality 42090) [19]. Damage types were assigned using the “Guide to Insect (and Other) Damage Types on Compressed Fossil Plants” [33]. Damage types are morphotypes defined by characters, such as size, shape, tissue alteration, damage pattern, and placement on the leaf, that are used to unambiguously categorize insect damage so that it can be quantitatively analyzed [34]. The number of insect DTs is positively correlated with the diversity of herbivorous insects that made the DTs across host plants in modern tropical rainforests in Panama, which supports the use of DTs for interpreting insect herbivore richness in the fossil record [35]. We reexamined all three Mexican Hat collections listed above for this study, and we rescored the USNM collection for damage (see Tables S1 and S2).

The Mexican Hat flora is dominated by *Platanus raynoldsii* (Platanaceae), *Juglandiphyllites glabra* (Juglandaceae), *Zizyphoides flabella* (Trochodendraceae), and *Cercidiphyllum genetrix* (Cercidiphyllaceae), which together comprise ca. 92% of the specimens [19]. There are 17 documented dicotyledonous angiosperm leaf species overall (Table 1), and one unknown monocot leaf species. The most common leaf species at Mexican Hat also are widespread throughout the Western Interior USA during the early Paleocene [14]. Fruits include the aforementioned *Polyptera manningii*; *Joffrea speirsii* (Cercidiphyllaceae), the presumed fruits of the local *C. genetrix* [36]; *Nordenskioldia* (Trochodendraceae), which has been correlated with *Zizyphoides* leaves [37]; and two unidentified fruits. Non-angiosperm taxa include leafy branches of the swamp redwood *Glyptostrobus europaeus* (Cupressaceae), which are very abundant but were not censused, and leaves of the fern *Onoclea hesperia* (Woodsiaceae).

**Table 1:** Angiosperm leaf species at Mexican Hat ranked by abundance in 2004 census [19].

Family	Species	Abundance in 2004 census	Percentage
Platanaceae	*Platanus raynoldsii* Newberry	1207	53.4%
Juglandaceae	*Juglandiphyllites glabra* Brown ex Watt	396	17.5%
Trochodendraceae	*Zizyphoides flabella* (Newberry) Crane, Manchester and Dilcher	231	10.2%
Cercidiphyllaceae	*Cercidiphyllum genetrix* (Newberry) Hickey	214	9.5%
Lauraceae	Lauraceae sp. 2 Wilf et al. 2006	87	3.9%
Unknown affinity	“*Populus*” *nebrascensis* Newberry	85	3.8%
Unknown affinity	Dicot 1 Wilf et al. 2006	12	<1%
Nyssaceae	*Browniea serrata* (Newberry) Manchester and Hickey	12	<1%
Unknown affinity	“*Ficus*” *artocarpoides* Lesquereux	4	<1%
Unknown affinity	cf. *Ternstroemites aureavallis* Hickey	2	<1%
Lauraceae	Lauraceae sp. 1	2	<1%
Unknown affinity	*Paleonelumbo macroloba* Knowlton	2	<1%
Unknown affinity	*Paranymphaea crassifolia* Newberry	2	<1%
Unknown affinity	Dicot 2 Wilf et al. 2006	1	<1%
Unknown affinity	Dicot 3 Wilf et al. 2006	1	<1%
Unknown affinity	Dicot 4 Wilf et al. 2006	1	<1%

Mexican Hat insect damage morphology and composition were compared to those of the Cretaceous Hell Creek and early Paleocene Fort Union Formation collections from the nearby Williston Basin in southwestern North Dakota, the Powder River Basin in southeastern Montana, and from early late Paleocene (58–59 Ma) collections available from the Bighorn Basin in north-central and the Greater Green River Basin in southwestern Wyoming (Table 2). From North Dakota, KRJ sampled fossil floras intensively across the K-Pg boundary in a 183 m composite section representing ca. 2.2 million years of deposition, as detailed in several papers [6]–[8], [38]. Subsequently, a total of 13,441 of the resulting museum specimens, from 106 stratigraphic levels, were scored for insect damage [11], and these are re-examined here, 9292 from the Cretaceous and 4149 from the Paleocene. These specimens are housed at DMNH and YPM. Collections made by DJP from early to middle Paleocene strata in the Williston Basin in North Dakota (2916 specimens at YPM [14], [39]; 66.0–58.2 Ma, calculated with time scale from [31] and sedimentation rates from [40]), as well as fossils from 14 early Paleocene sites in the Powder River Basin in Montana (1280 specimens at DMNH and YPM [39]; 65.1–61.8 Ma, calculated with time scale from [31] and sedimentation rates from [30]) were examined for insect damage for the first time in this study. For broader regional and temporal context, the leaf fossils from early late Paleocene localities in Wyoming (59.0–57.5 Ma) collected by PW and CCL and scored for insect damage in [19] were also reexamined. Voucher specimens preserving all insect damage types found on all host plants from field-census tallies at these Wyoming sites are curated at USNM and include 648 specimens from the Greater Green River Basin (USNM localities 41687 (*Persites* Paradise), 41691 (Kevin's Jerky), and 41694 (Haz-Mat)), and 440 specimens from Polecat Bench in the Bighorn Basin (USNM localities 42041 (Skeleton Coast) and 42042 (Lur'd Leaves)), as described in reference [19].

**Table 2:** Collections and repository information.

Formation	Basin	State	Age (Ma)	Localities	Specimens	Repository	Citation
Fort Union	Bighorn Basin	WY	59.0 and 57.5	2	440	USNM	Wilf et al., 2006
Fort Union	Greater Green River Basin	WY	59.0	3	648	USNM	Wilf et al., 2006
Fort Union	Powder River Basin	MT	65.1–61.8	14	1280	DMNH, YPM	Peppe, 2009; Peppe et al. 2011
Fort Union	Powder River Basin	MT	65	1	1073	DMNH, USNM, YPM	Williams, 1988; Lang, 1996; Wilf et al., 2006
Fort Union	Williston Basin	ND	66.0–58.2	102	7065	DMNH, YPM	Johnson, 2002; Peppe, 2010
Hell Creek	Williston Basin	ND	67.0–66.0	106	9292	DMNH, YPM	Johnson, 2002

We described all insect damage at Mexican Hat by host plant (except for previously described agromyzid mines [26]) and focused on leaf mines in the analysis, because they preserve morphological features that allow for detailed comparisons. These morphological features include [41], [42] 1) overall shape, size and length of the mine; 2) oviposition site structure; 3) changes in mine and frass trail width; 4) position of the mine on the leaf; 5) overall mine trajectory; 6) features of the mine border where it contacts unconsumed leaf tissue; 7) degree of restriction of the mine based on vein rank; 8) frass type, whether fluidized or particulate, and if particulate, pellet structure; 9) frass-trail continuity, including the presence of sap-feeding (fluidized frass) and whole-tissue consumption (particulate frass); 10) type of foliar tissue mined; and 11) size, shape, and structure of the terminal chamber, including whether an emergence hole or slit is present. Generalized DTs, such as hole feeding and margin feeding, are made by a variety of insect orders and cannot usually be attributed to specific insect herbivores; thus, their low extinction across the K-Pg is not likely to be biologically meaningful [11], although Carvalho et al. [35] found a decrease in host-specificity for these damage types. Gall-inducing insects are typically host-specialized, but morphological characteristics of galls on compression fossils are sometimes obscured, often because their three-dimensional structure is flattened, making detailed comparisons difficult. Mines are made by four orders of living insects: Coleoptera, Diptera, Hymenoptera, and Lepidoptera, and they can often be attributed to order, or even family [26], [41], [43]–[45]. In this study, the morphologies of leaf mines from all examined collections (Table S3) were compared to determine whether they were unique, among known fossil collections, to the Mexican Hat leaf-mining fauna.

We also focused on well-sampled surviving plant families to determine whether they provided refuge for insects after the K-Pg extinction. We compared insect damage on twenty-four Platanaceae species from the Hell Creek Formation (1397 specimens) to that on Paleocene *Platanus raynoldsii* (933 specimens; 724 from local early Paleocene; 214 from Mexican Hat, 41 from late Paleocene, WY). Also, we compared insect damage on five Cercidiphyllaceae species (323 specimens) from the Hell Creek Formation to Paleocene *Cercidiphyllum genetrix* (556 specimens; 374 from local early Paleocene; 52 from Mexican Hat, 130 from late Paleocene, WY).

Macrophotographs were taken using a Nikon D90 camera at DMNH, USNM, YPM, and the Paleobotany Laboratory, Pennsylvania State University. Microphotography was performed in the Paleobotany Laboratory, Pennsylvania State University, using a Nikon DS-Ri1 camera mounted on a Nikon SMZ 1500 binocular microscope. Images were processed using Nikon NIS Elements v. 3 software. The Photoshop CS5 and CS6 Align and Blend functions were used to vertically composite images of fossils with uneven surfaces as needed. Composite images were carefully checked for artifacts. Adobe Camera Raw Editor was used to reversibly adjust white balance, temperature, contrast, etc. as needed on whole images.

### The Mexican Hat Insect Damage Fauna

Insect damage is presented by host plant, in decreasing rank leaf-abundance order as shown in the 2004 field census [19] (Table 1).

### *Platanus raynoldsii*

**Figure 1:**
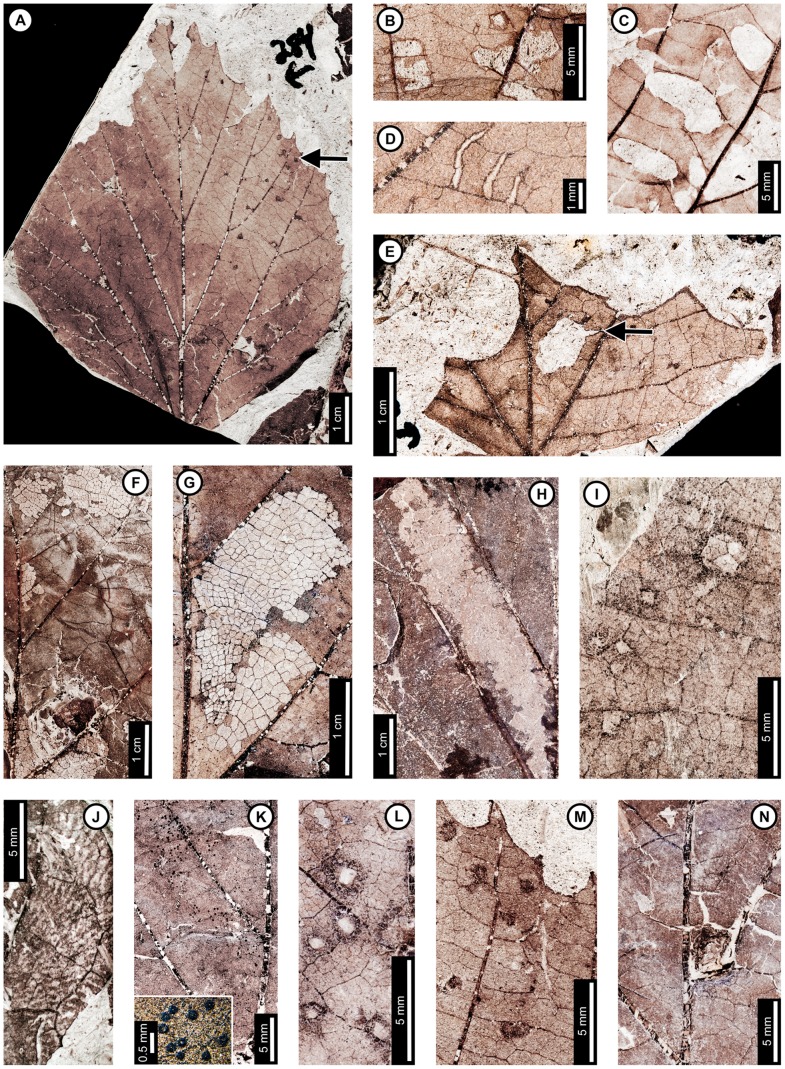
Non-mining insect damage on *Platanus raynoldsii* at Mexican Hat, early Paleocene, Montana. A: Margin feeding at leaf apex to midvein (DT15) and dark, circular galls on interveinal tissue (DT32; arrow expands to M) and adjacent to secondary veins (DT33; USNM 560105). B: Circular holes (DT2) and skeletonized areas (DT16) with well-developed reaction rims and minimal tertiary venation preserved (USNM 560106). C: Oval (DT4) and polylobate (DT3) holes bounded by tertiary veins (USNM 560107). D: Parallel-sided slots (DT8), which thin to a point (USNM 560108). E: Multiple margin feeding events characterized by shallow, semicircular incisions (DT12) and a large polylobate hole with possible secondary feeding event (arrow) on upper right side of the hole (DT5; USNM 560109). F: Skeletonized areas lacking reaction tissue (DT16) and crossing primary veins at the leaf base (DT56; USNM 560110). G: Skeletonized tissue with reaction rim adjacent to secondary veins and preserving fifth order venation (DT17; USNM 560111). H: Rectangular skeletonized area (DT19) bounded by primary and secondary veins (USNM 560112). I: Circular surface-feeding areas (DT29) with darkened reaction rims (USNM 560113). J: Parallel-sided, alternating light and dark lines of unknown origin; possibly viral damage, parallel lines of surface feeding, or fungal damage (DT23; USNM 560115). K: Curvilinear rows of dark, circular piercing and sucking marks with central depressions (DT46). Detail of piercing and sucking marks provided in inset (USNM 560114). L: Galls with thickened outer rims surrounding unthickened tissue (DT11) positioned on intercostal tissue and adjacent to secondary veins (USNM 560116). M: Detail of galls in (A) (DT32; USNM 560105). N: Three-dimensionally preserved gall near intersection of primary and secondary veins (DT32; USNM 560117).

**Figure 2:**
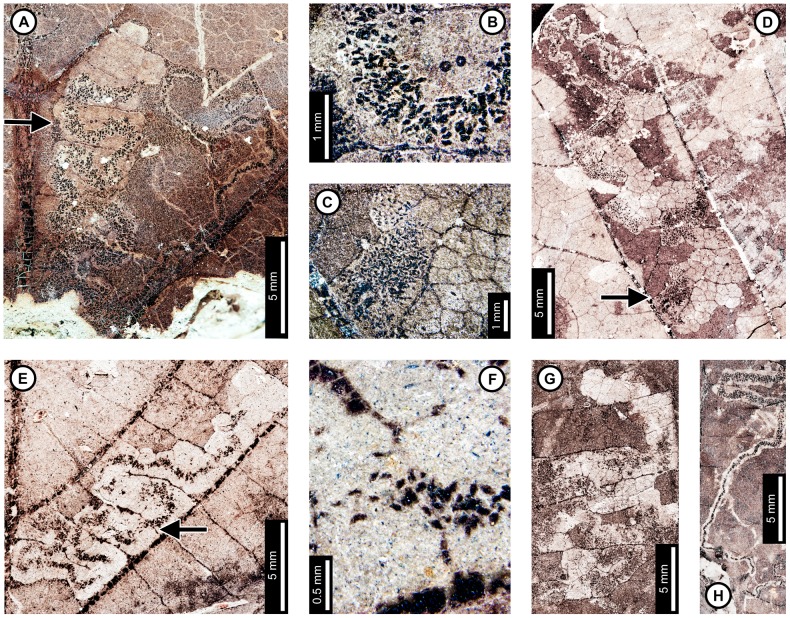
Association between *Platanus raynoldsii* and a lepidopteran leaf miner (DT91) at Mexican Hat. A: Near-complete leaf mine between two secondary veins (arrow expands to B; USNM 560118). B: Detail of terminal frass trail in (A) showing ellipsoidal pellets. C: Detail of frass trail in (D) showing ellipsoidal pellets packed near the margin of the expanded terminal mine. D. Complete mine with initial, gradually widening trail and expanded terminal path (USNM 560119; arrow expands to C). E. Mine delimited by secondary and tertiary venation (USNM 560120; arrow expands to F). F. Detail of frass trail in (E), showing ellipsoidal frass pellets. G: Mine with tightly coiled path and blotch-like appearance with ellipsoidal frass adjoining the mine margins (USNM 560113). H. Partially-preserved mine showing gradual widening during the early phase (USNM 498156).

**Figure 3:**
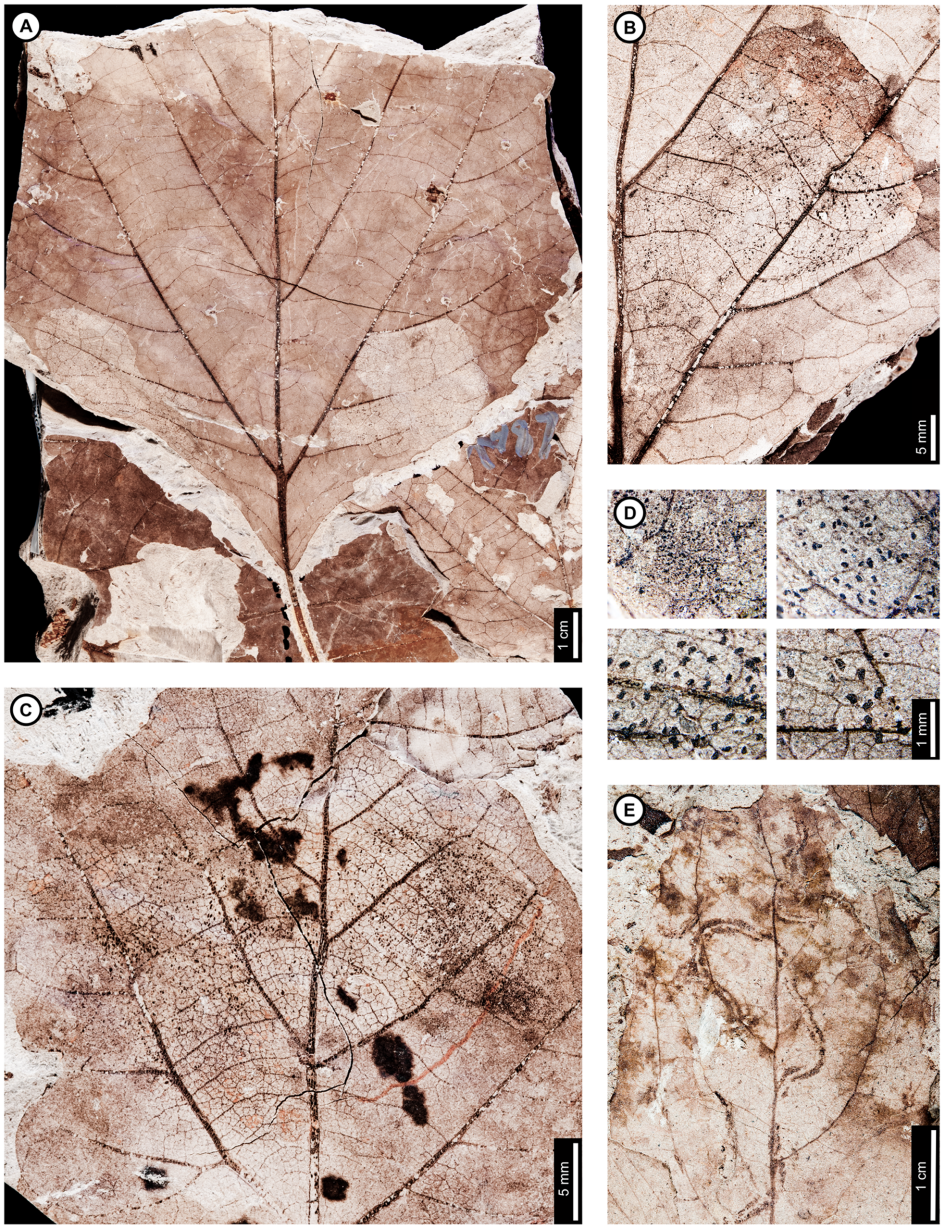
Sawfly (Tenthredinidae) blotch-mines (A–D; DT36) and a probable lepidopteran mine (E) on *Platanus raynoldsii*. A: Two blotch mines near the leaf base (USNM 498155). B: Closeup of blotch mine showing dispersed frass pellets (USNM 560121). C: Closeup of blotch mine on probable young leaf, showing increase in frass size from right to left (USNM 560122). D. Detail of frass pellets from the left blotch mine in (A). Each box depicts a different frass size, starting from the smallest (1^st^ instar; upper left box) to largest (4^th^ instar; lower right box) (USNM 498155). All four pictures are at the same scale for size comparison. E: Probable serpentine mine with parallel-sided frass trails (DT282; YPM 65939A).

Insect damage for this host plant is illustrated in Figures 1–3, excepting previously described agromyzid mines [26]. External foliage feeding on *P. raynoldsii* includes hole feeding, margin feeding, skeletonization, and surface feeding (Fig. 1). Circular holes (DT1, DT2 of [33]) range in diameter from 0.67–4.85 mm (Fig. 1B). Oval holes (DT4) average 7.56 mm long by 2.50 mm wide and are bounded by tertiary veins (Fig. 1C). Polylobate holes (DT3, DT5) range in length from 3.60–8.95 mm and 2.10–6.15 mm in width (Fig. 1E). Elongate holes include curvilinear, parallel-sided slots, which thin to a point (DT8; 1.15–2.55 mm L×0.21–0.36 mm W; Fig. 1D); reaction rims are typically 0.07 mm wide. Margin feeding damage is also present, including semicircular incisions across primary veins (DT12), ranging from 7.20–12.63 mm wide and 1.30–4.61 mm deep (Fig. 1E), and incisions extending towards the midvein (DT15) measuring 1.20–3.59 mm in width and 11.5 mm deep (Fig. 1A). Reaction rims associated with margin feeding range in width from 0.30–0.95 mm.

*Platanus raynoldsii* has five skeletonization DTs, typically in circular or oval patterns with no reaction tissue (DT16; Fig. 1F). When reaction tissue is present along the edges of skeletonized areas, it averages 1.16 mm in width (DT17; Fig. 1G). Rectangular patches of skeletonized tissue (DT19; Fig. 1H) typically cross major veins, but some are also bounded by major veins (between the midvein and two secondary veins). These rectangular patches of skeletonized tissue resemble damage made by extant leaf-rollers or tiers [46]. Skeletonized tissue that crosses primary veins at the base of the leaf is also present (DT56; Fig. 1F). Fourth and even fifth order venation is typically preserved in skeletonized areas of *P. raynoldsii* (Fig. 1F,G, H). Circular surface feeding marks measure 0.77–2.22 mm in diameter, with reaction rims ranging from 0.16–0.39 mm wide (DT29; Fig. 1I). A pattern of thin millimeter-wide lines alternating in color from light to dark is present on some specimens (DT23; Fig. 1J). The cause of this damage is unknown, but it may represent viral damage [47], fungal marks, or parallel surface feeding traces.

Circular piercing and sucking marks with central depressions (DT46; Fig. 1K) on *P. raynoldsii* range in diameter from 0.08–0.17 mm and are clustered in circular groups and curvilinear rows.

Three gall DTs are present on *P*. *raynoldsii*. Circular galls with thickened outer rims surrounding unthickened tissue (DT11; Fig. 1L) are positioned on intercostal regions and also adjacent to primary and secondary veins. The DT11 gall diameters range between 1.59–2.37 mm, and the unthickened inner tissue ranges from 0.50–0.75 mm. Other DTs include indistinct, dark, circular galls positioned on intercostal tissue and primary veins (DT32, DT33; Fig. 1A,M); these galls lack the unthickened inner region that characterizes DT11 and range in diameter from 0.84–1.50 mm. A three-dimensionally preserved circular gall (DT32; Fig. 1N) is positioned at the intersection of a primary vein and a secondary vein, and it measures 4.3 mm in diameter.

At least three and probably four mine DTs appear on *P. raynoldsii*. The previously described *Phytomyzites biliapchaensis* [26] is an Agromyzidae (Diptera) leaf mine characterized by an irregular to serpentine or linear path with a central or more typically an alternating frass trail composed of fluidized specks or segments, whose only, and abundant, occurrence is at Mexican Hat on *Platanus* leaves.

The second mine DT (Fig. 2) was previously assigned to DT91 [19]. The mines are characterized by a serpentine path with a widened terminal section and ellipsoidal frass pellets. Frass typically increases in size through the mine's course, ranging from 15–130 µm length and 10–75 µm width. The initial portion of the mine is serpentine, usually arising in intercostal tissue. It ranges in width from 0.32–0.37 mm, and frass fills the central 41–71% (Fig. 2). The frass pellets are initially spheroidal and measure 15 µm in diameter (min  = 11, max  = 20). The serpentine path then continues to widen, reaching 0.66–1.59 mm with frass filling the middle 65%. In most specimens, the mine increases greatly in width during the terminal phase (Fig. 2A, D). The width increase takes two forms: a winding path between tertiary veins bounded by primary or secondary venation (Fig. 2A, E) or a large expansion into a blotch-like mine, with clusters of frass deposited near the mine margins (Fig. 2D, G). Mine paths of the first terminal phase type (a winding pattern between tertiary veins) range from 1.53–2.16 mm in width and are controlled by tertiary venation. Frass fills the center 38–77% of the mines, and frass trails are 0.83–1.19 mm wide. Frass pellets are ellipsoidal and average 0.095 mm in diameter (min  = 0.069, max  = 0.13 mm) by 0.052 mm (min  = 0.038, max  = 0.073). The larvae that produced the winding paths between tertiary veins removed tertiary venation at crossing points (Fig. 2F). Mine paths of the second terminal phase type (elongate blotch-like mine) are 2.22–7.89 mm wide. Secondary veins bound the widest portions (Fig. 2D, G). The frass trails in serpentine sections of the mines are usually continuous but discontinuous in the wider, blotch-like portions (Fig. 2D, G). Frass usually is deposited in clusters near the mine margin (Fig. 2C). Mine courses are strongly influenced by primary, secondary, and tertiary venation throughout their length. Early portions of the mines tend to turn with primary and secondary veins and cross these major veins only where they are thin. Veins delimit the margins of mines, giving them a wavy appearance. There is no reaction tissue on any of the examined specimens.

The third mine DT on *P. raynoldsii* is a blotch mine assigned to DT36 (Fig. 3) [19]. There are five specimens with this association, including a large leaf with two mines, one on each side of the midvein and bordering the leaf margin (Fig. 3A), another large leaf with one mine that is positioned between the midvein and a first secondary vein, crossing a lateral primary vein (Fig. 3B), and a small, possibly young leaf with one mine crossing the midvein and present throughout a majority of the leaf blade (Fig. 3C). Frass pellets are ellipsoidal and dispersed throughout the mines. There are four discrete frass sizes presumed to represent instar growth phases, and similar sizes are clustered together (Fig. 3D). The smallest frass pellets are spheroidal (51 µm in diameter, standard deviation of ±6 µm) and are packed close together. The second, third, and fourth sizes are ellipsoidal and measure 133±9 µm L×74±7 µm W, 174±12 µm L×95±10 µm W, and 239±29 µm L×148±15 µm W, respectively. The general path of the larvae can be observed by following the width increases, and the distance between frass pellets tends to increase with frass pellet size. Major veins tend to control mine margins, but some portions end in interveinal tissue. There is no reaction tissue along the mine margins.

A probable mine is found on a poorly preserved, fragmentary leaf fossil of *P*. *raynoldsii* and is defined by a serpentine path with darkened margins, representing either reaction tissue or frass (DT282; Appendix S1; Fig. 3E). The mine varies in width from 1.10–1.80 mm, and there is no apparent widening trend. The darkened marginal areas vary in width from 0.33–0.84 mm and comprise around 70% of the overall mine width. Mine path and margins are not influenced by venation except for short sections along the midvein, which suggests that the mine was embedded in epidermal tissue. The beginning and end of the mine are not preserved.

### *Juglandiphyllites glabra*

**Figure 4:**
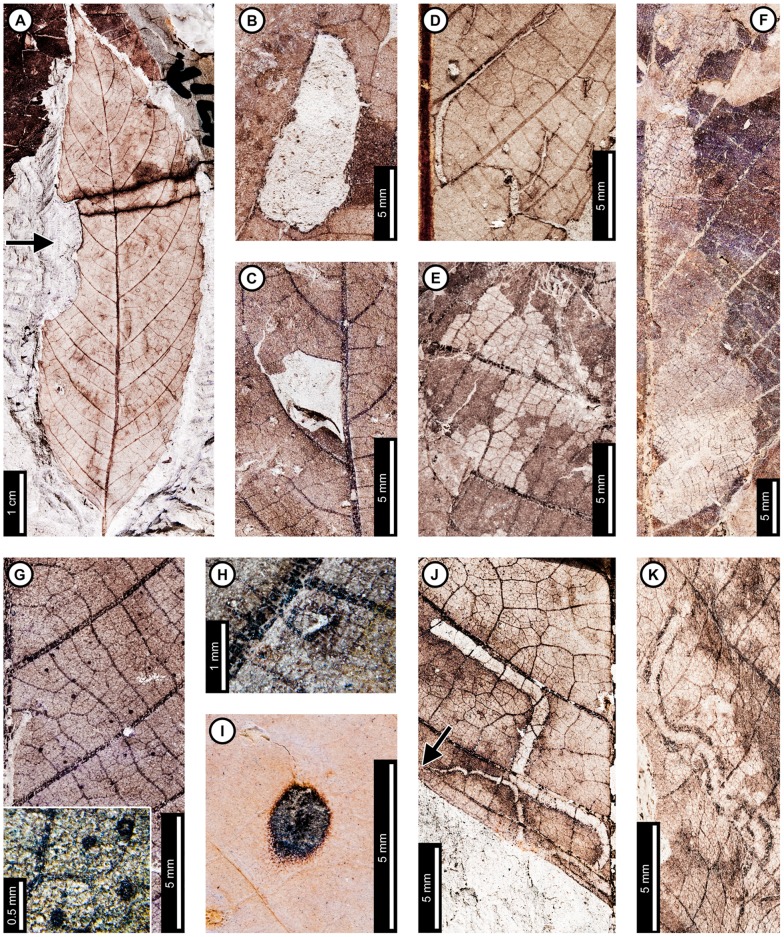
Insect-feeding damage on *Juglandiphyllites glabra* at Mexican Hat. A: Shallow, semicircular margin feeding (DT12; arrow; USNM 560123). B: Oval hole (DT4) delimited by secondary and tertiary veins (USNM 560124). C: Polylobate hole (DT5) bounded by primary, secondary, and tertiary veins (USNM 560125). D: Parallel-sided, elongate slot feeding, influenced by major veins (DT8), or possibly an abiotic tear (DMNH 35833). E: Patches of skeletonized tissue lacking reaction rim (DT16; USNM 560126). F: Rectangular skeletonized area (DT19; USNM 560127). G: Piercing and sucking marks with central depressions (DT46). Detail of marks in inset (USNM 560128). H: Gall with thickened outer rim and possible exit hole at the center (DT11) (USNM 560129). I. Gall on interveinal tissue (DT32) characterized by a darker outer rim and center (USNM 560130). J: Serpentine mine lacking frass, with a gradual width increase and strong association with major veins (DT105). Arrow marks the oviposition site (USNM 498159). K: Probable mine with parallel frass trails (DT282; YPM 65939).

**Figure 5:**
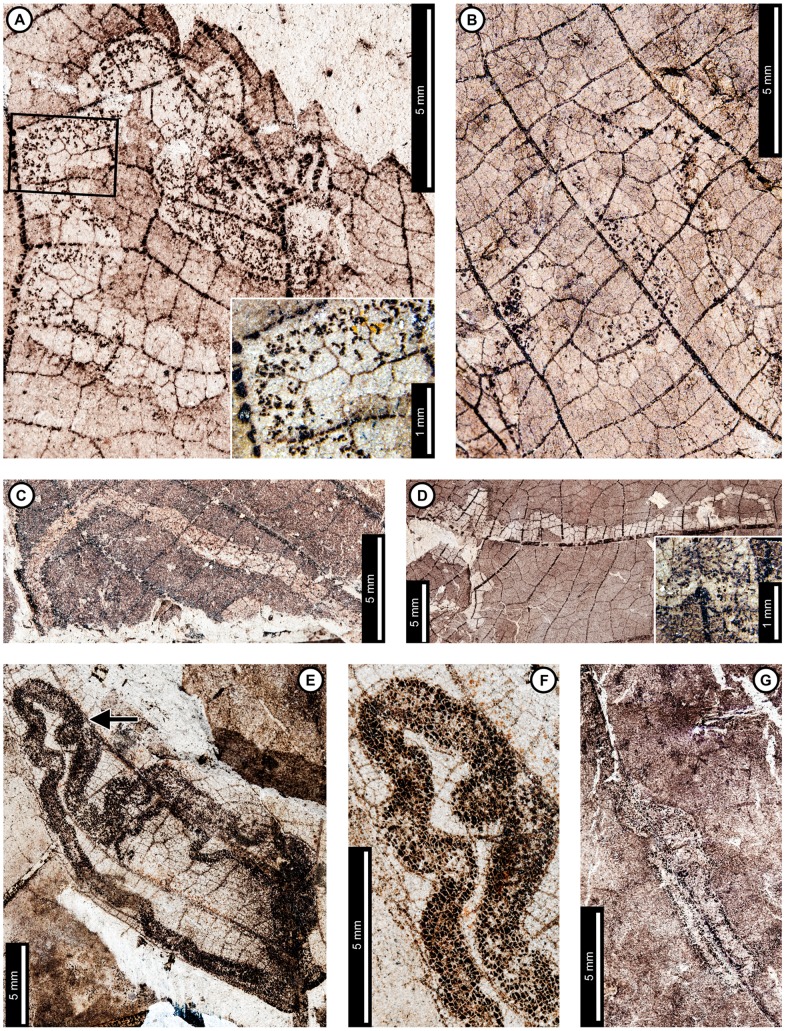
Additional mines on *Juglandiphyllites glabra* at Mexican Hat. A: Intestiniform mine with initially tightly packed frass trail and subsequent looser deposition of spheroidal frass pellets (DT91). Box indicates area expanded in the inset, showing detail of the frass trail (USNM 560131). B: Sinusoidal mine with loosely packed frass trail (DT91; USNM 560132). C: Linear mine with loosely packed frass trail (YPM 65888). D: Serpentine mine with sparse, spheroidal frass pellets and gradual width increase (YPM 65848). Detail of frass trail from counterpart in inset E: Sinusoidal mine packed with spheroidal frass pellets (DT92; arrow expands to F; USNM 498157). F: Detail of frass trail in (E), showing densely packed frass along the margins, and slightly looser frass in the center of the mine. G: Mine following secondary venation, with small, spheroidal frass pellets packed along mine margins (DT92; USNM 560124).

**Figure 6:**
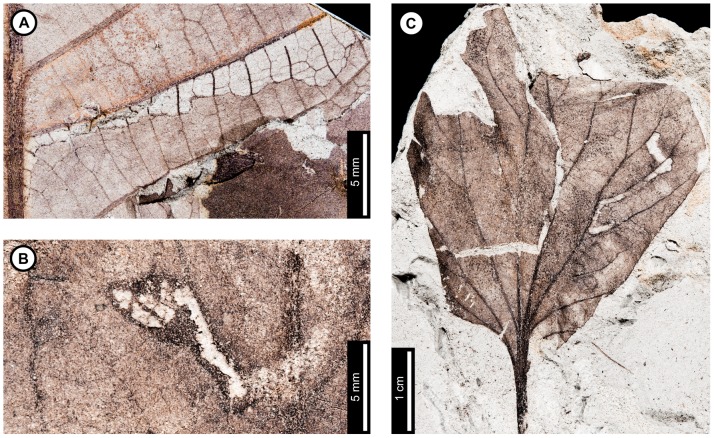
Damage type 42 mines from Mexican Hat and the latest Cretaceous of North Dakota, USA. A: Dramatically expanding mine, following primary and secondary venation, with ragged margins on *Juglandiphyllites glabra* at Mexican Hat (USNM 560133). B: Short, probable mine, with initial linear phase and expanded terminal chamber on *Liriodendrites bradacii* (Battleship – DMNH loc. 900; DMNH 7317). C: Probable mine with gradual width increase and fairly smooth margins on HC32 (Terry's HCIIa Site – DMNH loc. 2097; DMNH 19984).

Insect damage for this host plant is illustrated in Figures 4–6. Because *Polyptera manningii* and *Juglandiphyllites glabra* at Mexican Hat together represent the oldest reliable evidence for Juglandaceae [24], [25], this study likewise documents the earliest evidence for herbivory on Juglandaceae. *Juglandiphyllites glabra* includes the greatest number of distinct mine associations (at least four and probably five; all probable lepidopteran mines) of any plant species at Mexican Hat.

External foliage feeding on *J. glabra* (Fig. 4) includes hole feeding, margin feeding, and skeletonization. Circular and oval holes (DT1, DT2, DT4; Fig. 4B) range in diameter from 0.50 to 13.48 mm, with reaction rim widths averaging 0.17 mm. Polylobate holes (DT3, DT5; Fig. 4C) include one measuring 3.79 mm in length and bounded by primary, secondary, and tertiary venation. One specimen has elongate, parallel-sided slots with smooth margins (DT8; Fig. 4D); the slots vary in width from 0.20–0.57 mm and are partly influenced by secondary and tertiary venation. Margin feeding was only found on one specimen of *J. glabra* (Fig. 4A) and is characterized by two adjacent shallow, semicircular incisions along the margin (DT12) measuring around 10 mm wide by 4.5 mm deep, with a 0.09–0.60 mm wide reaction rim. Only two specimens were found to have skeletonization: one with patches of skeletonized tissue, with no reaction rim and fifth order venation preserved (DT16; Fig. 4E), and another specimen exhibiting a rectangular, elongate skeletonized area (DT19; Fig. 4F) following the midvein. The skeletonized area decreases in width apically, starting at 11.76 mm and decreasing to 2.52 mm, before a sudden width increase to 7.34 mm. The portion closest to the leaflet apex is not preserved. Wide, elongate skeletonization is typical of lepidopteran leaf-roller or tier larvae [46], and the changes in width may be related to instar development.

Piercing and sucking marks are found throughout two leaflets (Fig. 4G). The marks are dark and circular with a central depression (DT46) and are randomly distributed. They range in diameter from 0.18–0.24 mm.

Two *J. glabra* leaflets were found with galls. One gall is circular with an outer rim of thickened tissue (DT11; Fig. 4H) measuring 0.50 mm in diameter. Leaflet venation is visible in the center of the gall. The gall measures 0.33 mm in diameter. The second specimen (Fig. 4I) has a single, dark, circular gall (DT32) measuring 1.0 mm in diameter. The outer rim is black and surrounds an inner gray area. Within the gray area in the center of the gall is a black region, possibly representing the chamber, similar in color to the rim. This gall is positioned on interveinal tissue. The galls on *J. glabra* are superficially similar to some galls on modern *Carya* (Juglandaceae) made by gall midges (Cecidomyiidae) [48].

Among the lepidopteran-type mines on *J. glabra*, one was previously assigned to DT105 [19] and occurs on a single specimen (Fig. 4J). The mine is serpentine and characterized by parallel sides, angular turns, a semicircular terminus, and a lack of frass. The miner may have been a sap-feeder, i.e. a consumer of cell protoplasts and not the cell walls that frequently are processed as fecal pellets. The oviposition site is next to a basal secondary vein (Fig. 4J, arrow). The initial portion is 0.19 mm wide and follows a serpentine path before following the basal secondary vein. It crosses the basal secondary vein near the primary vein, follows the primary vein, and then turns along the next secondary vein. The mine then crosses the secondary vein and extends linearly across interveinal tissue. Finally, the mine meets the next secondary vein, and follows it to termination. There is no major width increase or evidence of a pupation chamber, so the mine may have been aborted. The mine path is greatly affected by major but not tertiary veins, and it usually turns and follows the veins at first contact. There is a steady width increase throughout the mine length, reaching 0.95 mm in width by the terminus. Mine margins are smooth, and there is a thin reaction rim measuring 0.15 mm surrounding the mines.

A second mining association on *J. glabra*, found on four specimens (Fig. 5A–D), was previously assigned to DT91 [19]. The mines are characterized by a serpentine path with loosely packed, and locally sparse, spheroidal frass pellets. The initial portion of the mines are serpentine or intestiniform and may have overlapping sections whereby the earlier path is occupied by later stages. The earliest frass trail is 0.21 mm wide on one specimen (Fig. 5A), but overall mine width in this portion is unknown because of overlapping mine paths. The earliest frass trail is also not preserved on the other specimens. Mine width increases very gradually throughout the course of the mine (min  = 0.38 mm; max  = 2.33 mm), but the width increase is most dramatic near the oviposition site (Fig. 5D). The subterminal frass trail varies from 0.29–1.13 mm wide. Frass trails are composed of individual spheroidal frass particles, filling 25–70% of the mine width and often adjoining the outside mine margin at turns. Frass does not change in size through the length of the mine (early frass diameter  = 53±9 µm; latest frass  = 51±8 µm). The terminal 2–3 mm of the mines contain no frass. All sections of the mine have wavy borders and no reaction tissue. Borders are controlled by tertiary venation in some areas, but the mines cross over both secondary and tertiary veins (Fig. 5A–D).

A third mining association on *J. glabra* was assigned to DT92 [19] (Fig. 5E–F). It is preserved on a fragmentary leaflet, although the mine is nearly complete. The mine is serpentine and fully packed with spheroidal frass pellets. It increases in width throughout, starting at 0.16 mm wide and terminating at 1.58 mm wide. Frass pellets fill the width of the mine, although frass is more densely packed near the margins than the center (Fig. 5F). The densely packed, marginal frass trails comprise about 25% of each side of the mine. Frass increases in diameter through the length of the mine (from 0.11 to 0.18 mm). Tertiary veins do not guide the mine, but it does follow the margin and the primary and secondary veins. The mine margin is smooth, and there is no reaction rim. A mine on another specimen of *J*. *glabra* (Fig. 5G) was also assigned to DT92, and it may represent a poorly preserved example of the same association (Fig. 5G). The early portion of the mine is serpentine, and it then follows a secondary vein in the direction of the leaf base. The mine then crosses the secondary vein and reverses direction, following the same vein distally. Frass pellets are spheroidal and measure 0.07±0.01 mm in diameter. No frass size difference is observed through the length of the mine. There is no reaction rim along the margins. Like the specimen in Fig. 5E–F, frass is packed densely on both sides of the mine. The mines differ in that there is no frass packed in the middle of the mine in the specimen shown in Figure 5G.

A fourth mine DT on *J. glabra*, assigned to DT42 [19], also lacks frass but differs from DT105 in that both tertiary and major venation affect the mine path and margins (Fig. 6A). The oviposition site is adjacent to the midvein. The mine dramatically widens to 0.72 mm and turns away from the primary vein, following the first intersecting secondary vein for the rest of the preserved portion, widening throughout to a maximum observed width of 3.83 mm. The mine terminus was not recovered. The mine margin located in the intercostal area is wavy and delimited by quaternary veins. There is no reaction tissue preserved.

A probable fifth mine DT on *J. glabra* is characterized by a serpentine path with two dark lines along the margins, representing reaction tissue or frass (DT282; Appendix S1; Fig. 4K). The mine is around 0.85 mm wide throughout, and there is no major width increase. The dark areas along the margin range in size from 0.19–0.37 mm, taking up 22–44% of the overall width of the mine. Leaf venation does not influence mine path, which suggests that the mine traveled through epidermal tissue. The proximal and terminal portions of the mine were not preserved.

### *Zizyphoides flabella*

**Figure 7:**
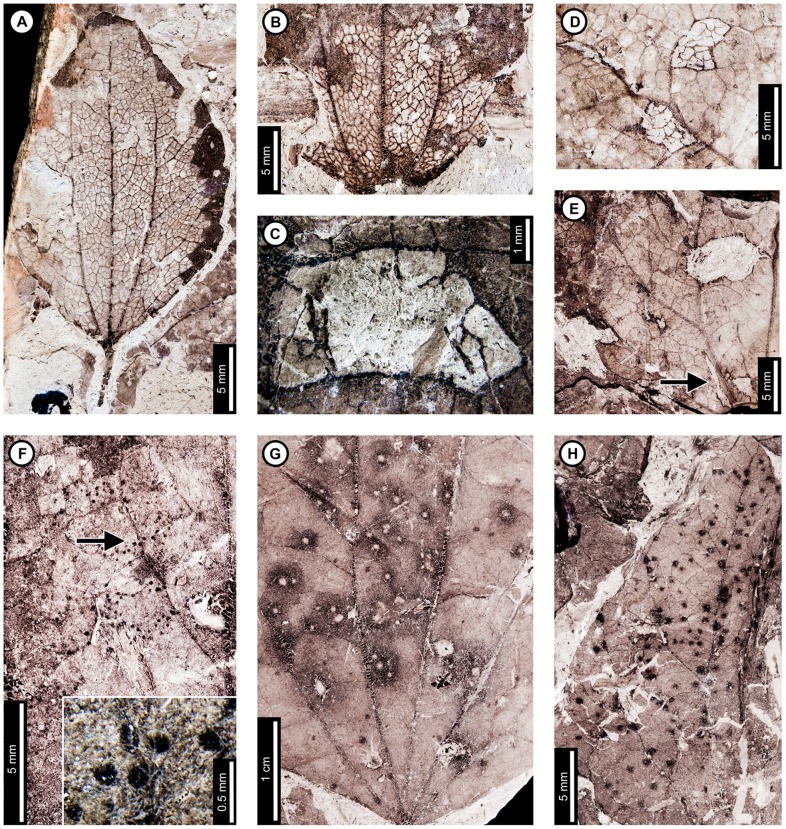
Non-mining insect damage on *Zizyphoides flabella* at Mexican Hat. A: A nearly completely skeletonized leaf lacking reaction tissue (DT16; USNM 560134). B: Skeletonization crossing primary veins at the leaf base (DT56; USNM 560135). C: Polylobate hole (DT5) delimited by tertiary venation and exhibiting uneaten veinal stringers (USNM 560136). D: Surface feeding (DT29) delimited by tertiary veins (USNM 560137). E: Polylobate holes (DT3, DT5) and an elongate hole lacking parallel sides (DT7; arrow) near the base. Also, note the small margin feeding incision (DT15) on the left side (USNM 560138). F: Curvilinear swaths of piercing and sucking marks with central depressions (DT46). Arrow indicates area expanded in the inset, showing detail of the piercing and sucking marks (USNM 560139). G: Galls with thickened outer rims and possible exit holes at the centers (DT11; USNM 560140). H: Dark, circular galls on interveinal tissue (DT32), primary veins (DT33), and secondary veins (DT34) (USNM 560141).

**Figure 8:**
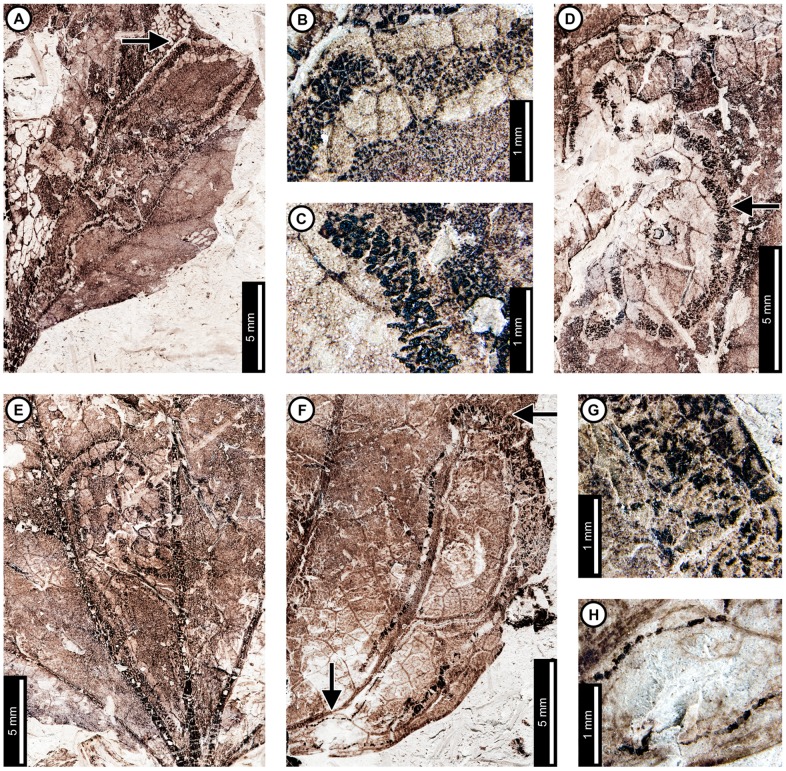
Leaf mines on *Zizyphoides flabella* at Mexican Hat. A: Serpentine mine delimited by primary veins, with gradual width expansion and centered frass trail packed with ellipsoidal pellets (DT91; arrow expanded in B; USNM 560142). B: Detail of later frass trail in (A), showing increase in frass size possibly associated with instar growth. C: Detail of frass trail in (D), showing tightly-packed, ellipsoidal frass pellets. D: Leaf mine with a central frass trail composed of ellipsoidal pellets (DT91; arrow expanded in C; USNM 560136). E: Leaf mine looping between primary veins, with central frass trail (DT91; USNM 560143). F: Threadlike mine (DT41), with gradually expanding width. Frass is initially densely packed and lacks individual pellets (lower arrow, expanded in H), but subsequently loosens into a wider trail (upper arrow, expanded in G) (USNM 498160). G: Detail of loosened frass trail in (F). H: Detail of early, threadlike path, with densely packed frass trail in (F).

Insect damage for this host plant is illustrated in Figures 7–8. We note that Lang (1996) censused insect damage on 295 specimens of *Z. flabella* at Mexican Hat and noted high levels of overall herbivory (48.5% leaves damaged), skeletonization (31.9%), and mining (3.1%). In the present study, external foliage feeding on *Z. flabella* (Fig. 7A–E) includes hole feeding, margin feeding, skeletonization, and surface feeding. Circular holes on *Z. flabella* range in diameter from 0.33–1.48 mm (DT1, DT2; Fig. 7E), with reaction rims ca. 0.18 mm wide. Polylobate holes range from 1.74–6.94 mm long by 2.22–4.21 mm wide (DT3, DT5; Fig. 7C, E, F), and they include one hole with uneaten veinal stringers (Fig. 7C). An elongate hole lacking parallel sides (DT7; Fig. 7E, arrow) appears adjacent to a primary vein, measuring 5.57×0.45 mm. Two margin feeding specimens are characterized by a polylobate incision into the margin (DT12), and an incision extending to a secondary vein measures 3.26 mm wide by 4.37 mm deep (DT15; Fig. 7E).

*Zizyphoides flabella* is associated with three skeletonization damage types. The most common of these are large skeletonized areas lacking reaction tissue (DT16; Fig. 7A), with third to fourth order venation intact. The veins of some specimens appear darker and thickened in skeletonized areas, suggesting a reaction to feeding (DT17). Skeletonization is also present at the bases of leaves (DT56; Fig. 7A, B), crossing all venation. Small patches of surface feeding (DT29; Fig. 7D) bound by tertiary venation are also present, with fourth order venation intact.

Piercing and sucking marks on *Z. flabella* are positioned in curvilinear rows. The marks are dark and circular and have central depressions (DT46; Fig. 7F). They measure 0.13 mm in diameter.

Four gall damage types are found on *Z. flabella* (Fig. 7G–H), including circular galls with dark, thickened tissue surrounding a lighter colored, circular area at the center (DT11), possibly representing exit holes. The internal area for these examples of DT11 measures 0.29–0.71 mm in diameter, and the overall gall diameter is 0.84–1.26 mm. Many galls are positioned close together with their thickened, outer tissues overlapping (Fig. 7G), and may actually be compound galls, consisting of multiple chambers within the same, structurally confluent gall. Black circular galls lacking a distinct rim are found on interveinal tissue (DT32) and against primary (DT33) and secondary veins (DT34); these galls have diameters of 0.34–0.71 mm. (Fig. 7H).

*Zizyphoides flabella* has two mining associations. The first was assigned to DT91 [19], and there are six specimens of this interaction (Fig. 8A–E). The mine is serpentine and typically oviposited near a primary vein. It follows the vein and loops around to the adjacent primary vein (Fig. 8A, D, E). Initial width is 0.45–0.60 mm, with a centered frass trail taking up 35–50% of the mine. Individual frass pellets are spheroidal–ellipsoidal and 0.045 mm in diameter. The width of the mine increases throughout, ending at 1.1–1.9 mm. The percentage of the mine taken up by frass tends to decrease with width, ranging from 30–40%, although the ratio does not change in some specimens. Frass size increases towards the end of the mine, ranging in length from 0.05–0.09 mm and width from 0.041–0.082 mm (average 0.057±0.01; Fig. 8B, C). The frass trail terminates at the end of the mine. The paths of the mines are affected by primary and tertiary venation, and veins border mine margins. Tertiary venation causes the mine margins to be wavy, and the width of the mine decreases when crossing tertiary veins (Fig. 8B). Reaction tissue width outside the mine margins measures 0.09 mm.

The second mine association on *Z. flabella* was assigned to DT41 [19], and only one specimen with this association is known (Fig. 8F–H). The mine is serpentine and initially threadlike, averaging 0.10 mm in width (Fig. 8H). It expands in width throughout its length, ending at 0.24 mm wide. The mine typically borders or crosses primary and secondary veins. The frass is non-particulate and fills the width of the mine, although frass appears to have been lost during preservation in some parts of the mine (Fig. 8H). The frass trail loosens near the leaf margin and widens to 0.95 mm (Fig. 8G), but it thins out again to 0.24 mm at the end. The margins are smooth throughout the length of the mine. The early, threadlike part of the trail is depressed from surrounding leaf tissues, suggesting that it was probably a full depth mine, with all the tissue between the upper and lower epidermises removed by the leaf miner. We note that Lang ([27]; his Figure 6.1.4 A–C) figured an apparently distinct blotch mine with ellipsoidal frass pellets on *Z. flabella*. Because we did not examine this specimen, it is not included in data analyses.

### *Cercidiphyllum genetrix*

**Figure 9:**
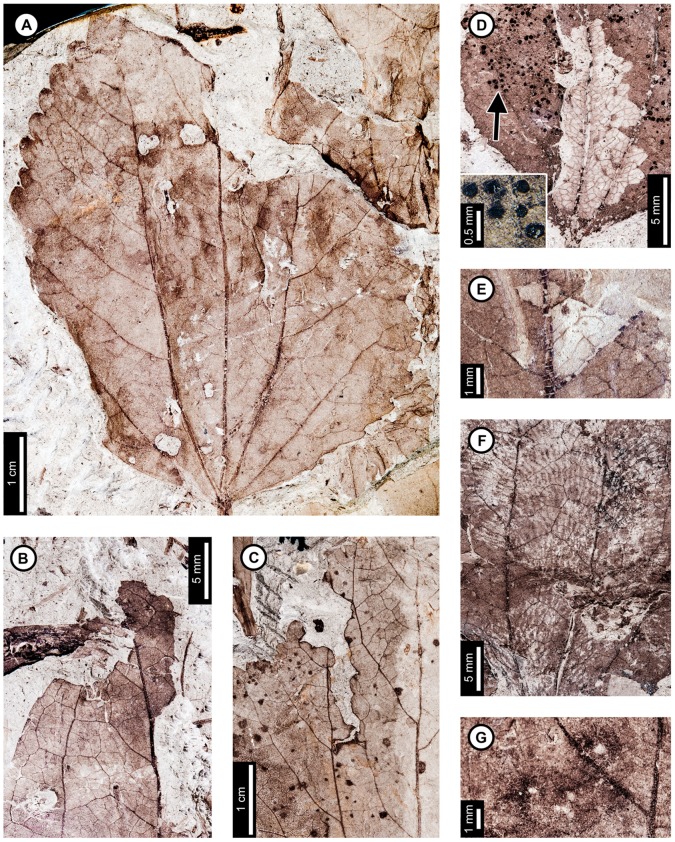
External foliage feeding on *Cercidiphyllum genetrix* at Mexican Hat. A: Circular (DT2) and polylobate holes (DT3) with thin reaction rims (USNM 560144). B: Semicircular margin feeding near leaf apex (DT12; USNM 560145). C: Deeply trenched margin feeding towards the midvein (DT15; possibly an abiotic tear) and dark, circular galls on interveinal tissue (DT32), primary veins (DT33), and secondary veins (DT34; USNM 560146). D: Skeletonized area at the leaf base (DT56) and circular piercing and sucking marks with central depressions spread throughout the leaf (DT46). Arrow indicates area expanded in the inset, showing detail of the piercing and sucking marks (USNM 560147). E: Surface feeding delimited by two secondary veins and crossing the midvein. Surface feeding crossing the midvein has polylobate reaction rim (DT30; USNM 560148). F: Alternating light and dark lines of unknown origin; possibly viral damage, parallel lines of surface feeding, or fungal damage (DT23; USNM 560149). G: Galls with thickened outer rims surrounding unthickened inner tissue (DT11; USNM 498161).

**Figure 10:**
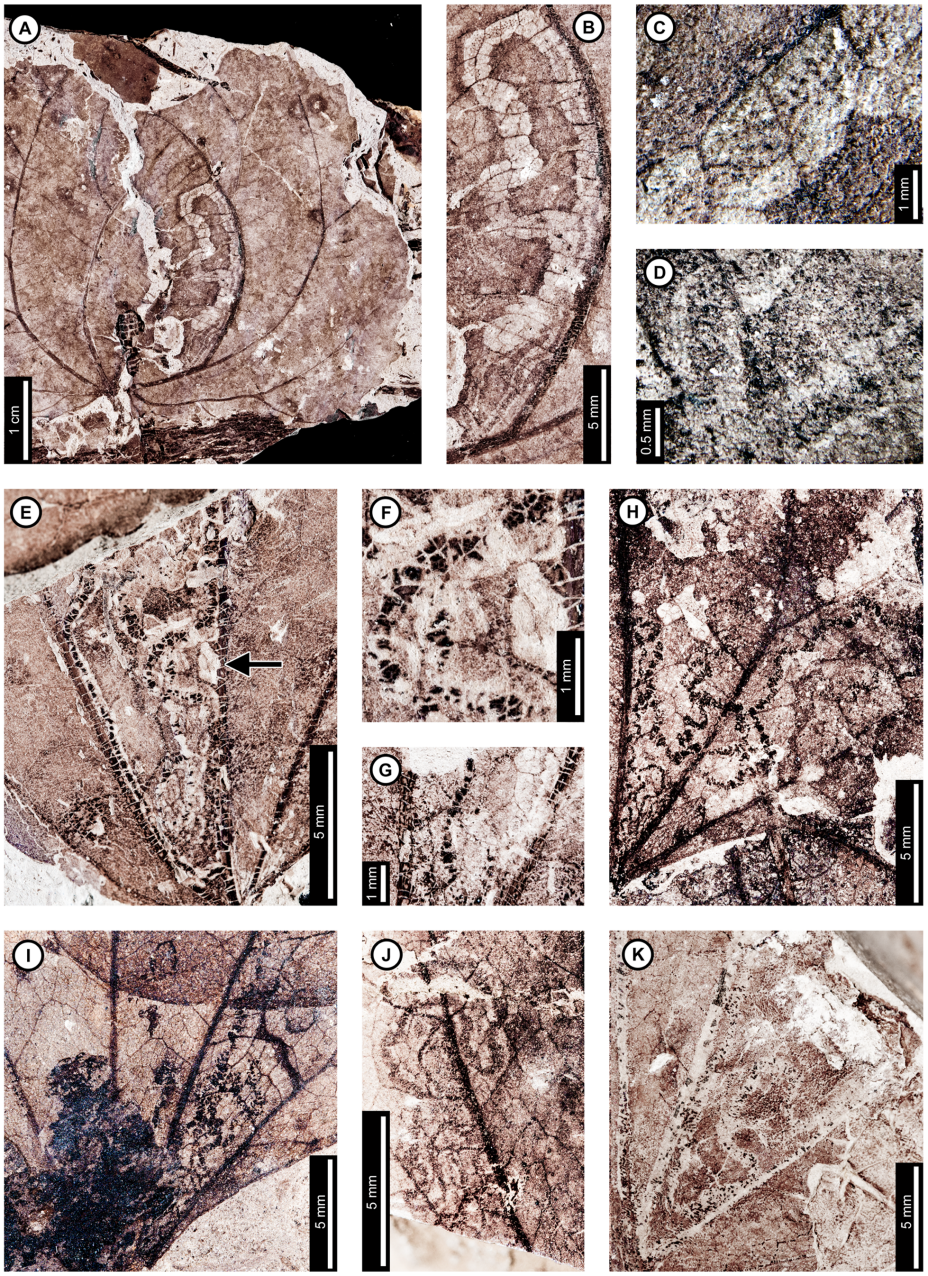
Leaf mines on *Cercidiphyllum genetrix* at Mexican Hat (A–G) and late Paleocene Wyoming localities (H–K). Mines on E–K (DT41) are interpreted as belonging to the same species of lepidopteran leaf miner, representing at least a 6 million year association between the host and miner. A: Mine characterized by overlapping trail, gradual width increase, spheroidal pellets in a frass matrix, and lack of frass in terminal portion (DT91; USNM 498161). B: Closeup of mine in (A). C: Closeup of frass trail in (A) and (B), showing spheroidal frass pellets in a darkened matrix. D: Detail of initial frass trail in (A) and (B). E: Serpentine mine characterized by initial threadlike phase arising from the base of a multi-veined leaf, widening path, smooth margins, and packed frass (DT41; arrow expanded in F; USNM 560150). F: Detail of frass trail in (E), showing tightly packed frass. G: Detail of counterpart of (E), showing packed frass in early portion of the mine. H: Threadlike mine with increasing width positioned at the base of the leaf (DT41) (Haz-Mat; USNM 560151). I: Threadlike mine with increasing width positioned at the base of the leaf between two primary veins (DT41) (Haz-Mat; USNM 560152). J: Tightly coiled mine with packed frass (DT41) (Skeleton Coast; USNM 560153). K: Poorly preserved mine positioned at the base of the leaf between two primary veins interpreted as DT41 (Skeleton Coast; USNM 560154).

Insect damage for this host plant is illustrated in Figures 9–10. External foliage feeding on *C. genetrix* includes hole feeding, margin feeding, and skeletonization (Fig. 9). Circular holes range in diameter from 0.65–3.36 mm (DT1, DT2; Fig. 9A). A single three-lobed hole measures 2.84 mm between the two most distant points (DT3; Fig. 9A). Reaction rims for all holes measure ca. 0.18 mm width. Margin feeding associations include a semicircular, shallow incision (DT12; Fig. 9B) measuring 8.62 mm wide and 3.43 mm deep, and a deep incision extending towards the midvein (DT15; Fig. 9C) measuring 6.76 mm wide and 21.25 mm deep.

Skeletonized tissues include circular areas with no reaction tissue (DT16) that are located between primary veins at the leaf base (DT56; Fig. 9D). Tertiary venation is visible, but higher venation was either removed by the insect's feeding or by taphonomic processes. Surface feeding with reaction tissue occurs at the intersection between a primary and secondary vein and across a primary vein (DT30; Fig. 9E). The damaged area on the primary vein is 1.56 mm in diameter and is surrounded by a polylobate reaction rim measuring 0.12 mm wide. There are also curved, parallel swaths that travel from the leaf base to the apex and diverge in opposite directions along a primary vein (DT23; Fig. 9F). The alternating light and dark lines measure 1 mm in width. The lighter lines have a white film on the grains, and some individual grains have both white and brown coloration. The cause of these marks is unknown, but they may represent viral damage [47], parallel surface feeding, or fungal damage.

The piercing and sucking marks on *C. genetrix* are typically dense and spread throughout leaf. They are characterized by circular punctures with a central depression, filled in with dark carbonized material (DT46; Fig. 9D). There are a variety of puncture sizes, ranging from 0.09–0.44 mm in diameter.

Circular galls characterized by a darkened outer rim, surrounding a lighter inner area representing unthickened tissue (DT11; Fig. 9G), are positioned on secondary veins and interveinal leaf tissue. The total diameter ranges from 0.80–1.13 mm, and the diameters of the inner area range from 0.13–0.24 mm. Indistinct, black circular galls are also observed on *C. genetrix*. The galls are positioned on interveinal tissue (DT32), primary veins (DT33), and secondary veins (DT34). They range in diameter from 0.45 to 1 mm (Fig. 9C).

*Cercidiphyllum genetrix* has two mining associations. The first is a serpentine mine known from one specimen, with a steady width increase from oviposition to terminus, previously assigned to DT91 [19] (Fig. 10A–D). The oviposition site was not preserved because a later portion of the mine passed through it. The mine is initially serpentine and affected by tertiary and higher-order venation. The earliest preserved stage of the mine is 0.28 mm wide, and the middle 62% of the mine is filled with sparse, spheroidal frass pellets in a lighter colored frass matrix (Fig. 10D). As the mine widens to 0.90 mm, individual spheroidal frass pellets are visible but spread far apart (Fig. 10C). The visible frass pellets average 33.0±4 µm in diameter. The individual pellets are surrounded by a lighter colored frass matrix, possibly fluidized frass, which is similar in appearance to the early frass trail. The percentage of the mine filled by frass decreases to 43% in the widest portions of the mine (Fig. 10B). The end of the frass trail is semicircular, and the last 22 mm of the mine contains no frass, except for that left over from earlier phases of the mine that were overlapped. Early portions of the mine are smooth-sided and parallel. Quaternary veins delimit later portions of the mine, making the margins wavy (Fig. 10C). Primary and secondary veins also bind the mine margins in some areas. The mine does not cross primary or secondary veins, but it does cross tertiaries. Darkened reaction tissue, measuring up to 0.87 mm in width, borders the outside margins of the wider portion of the mine.

A second leaf mine association, assigned to DT41 [19], is positioned between two primary veins at the leaf base of one specimen (Fig. 10E–G). The early mine phase averages 0.27 mm in width and widens to 0.92 mm by the latest preserved portion. The mine path is serpentine, and frass fills 32–65% of the width of the mine (Fig. 10F). The frass trail is broken into irregular pieces, which may be an artifact of preservation, possibly caused by cracking and fragmentation of the frass trail upon desiccation of the leaf. The mine margins are smooth, and there is no reaction tissue along their outside. The mine does not cross primary veins and does not appear to be directed by higher venation. On the counterpart, the frass trail appears to expand to the entire width of the mine, but the preservation is poor (Fig. 10G). The complete mine path is not preserved because of fracturing. The base of the leaf is preserved on the part, and the counterpart preserves the base and apex. Both are missing the center portion of the leaf.

### Lauraceae species

**Figure 11:**
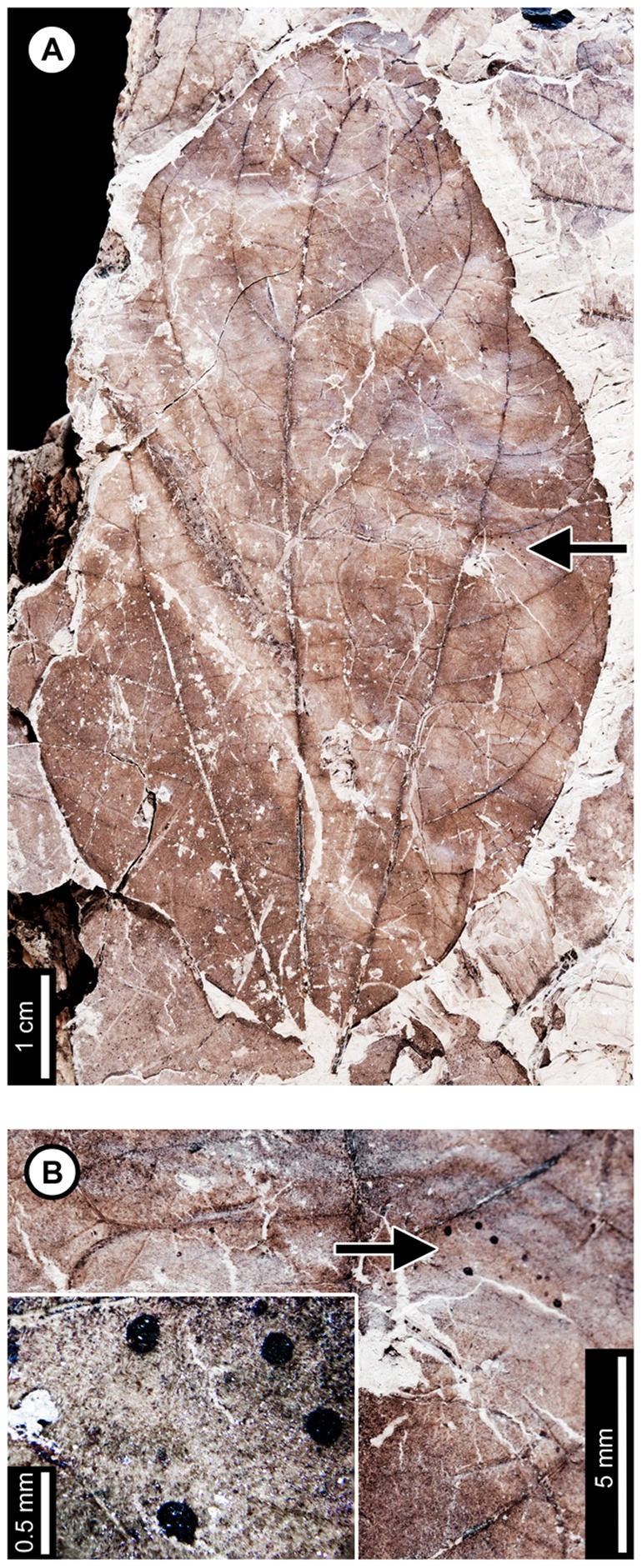
Insect damage on Lauraceae sp. 1 at Mexican Hat. A: Piercing and sucking marks with central depression (DT46; arrow expanded in B; USNM 560155). B: Closeup of cluster of piercing and sucking marks (DT46) on (A). Arrow indicates area expanded in the inset, showing detail of piercing and sucking marks.

Insect damage for Lauraceae species 1 is illustrated in Figure 11. This host has numerous piercing and sucking marks. The marks are dark and circular with a central depression (DT46; Fig. 11A–B) and are clustered in a small group. They range in diameter from 0.15–0.24 mm.

**Figure 12:**
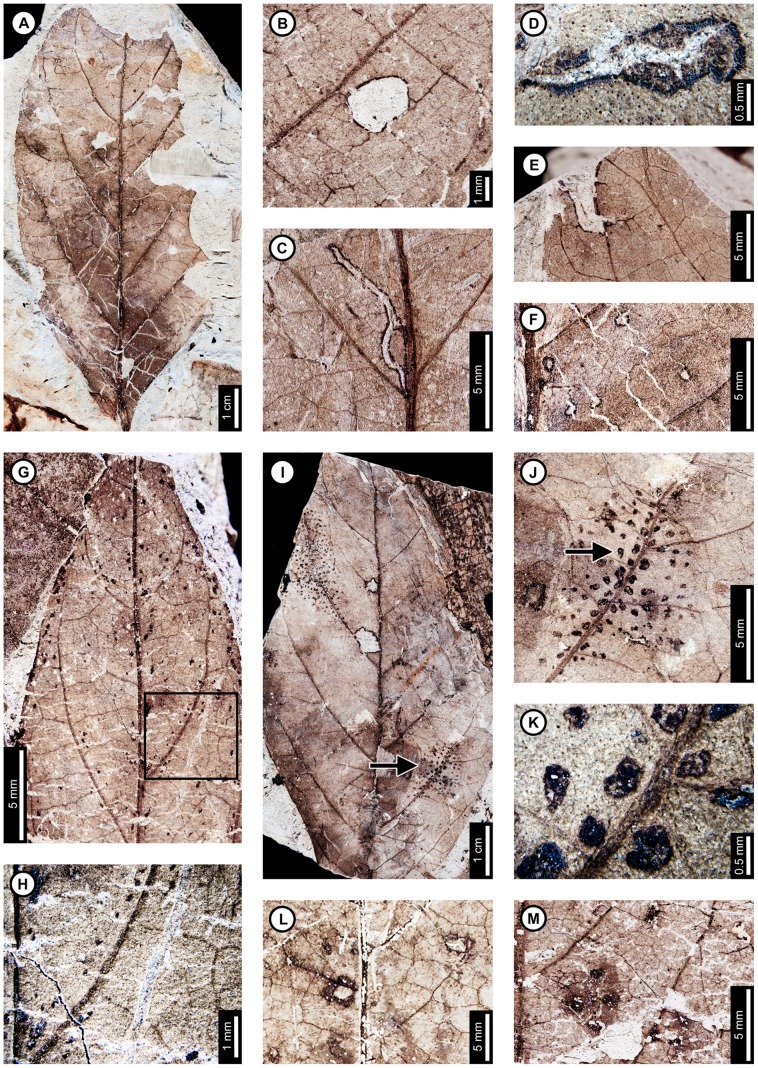
Insect damage on Lauraceae sp. 2 at Mexican Hat. A: Shallow, semicircular margin feeding along the sides of the leaf (DT12) and at the apex through the midvein (DT13) (USNM 560156). B: Circular hole (DT2) near secondary vein (USNM 560157). C: Elongate, parallel-sided hole (DT8) positioned at the intersection between the primary and secondary veins (USNM 560158). D: Elongate and tapering hole (DT7), with well-developed reaction tissue (USNM 560159). E: Margin feeding incision along a secondary vein towards the midvein (DT15; USNM 560160). F: Surface feeding areas (DT29) with well-developed reaction rims (USNM 560161). G: Elliptical and ovoidal piercing and sucking marks (DT48) associated with major veins and leaf margins (box expanded in H; USNM 560162). H: Detail of piercing and sucking marks in (G), showing linear association with secondary veins. I: Elliptical groupings of piercing and sucking marks (DT281) associated with secondary veins (arrow expanded in J; USNM 560163). J: Closeup of a piercing and sucking cluster in (I), showing an increase in the size of individual marks towards the vein (arrow expanded in K). K: Detail of piercing and sucking marks in (I) and (J). L: Galls with thickened outer rims surrounding unthickened tissue (DT11; USNM 560164) M: Circular galls clustered in groups of four and dispersed randomly on interveinal tissue (DT32) and adjacent to secondary veins (DT34; USNM 560157).

Insect damage for Lauraceae species 2 is illustrated in Figure 12. Lauraceae species 2 exhibits hole feeding, margin feeding, surface feeding, piercing and sucking, and galling. No leaf mines have been recorded. Circular holes are 0.69–2.47 mm in diameter (DT1, DT2; Fig. 12B) with reaction rims ranging in width from 0.07–0.18 mm. Polylobate holes range in length from 1.81–5.24 mm long (DT3, DT5; Fig. 12I) with 0.14–0.22 mm wide reaction rims. There are also parallel sided, curvilinear slot feeding marks (DT8; Fig. 12C), ranging in length from 3.10 to 9.39 mm and in width from 0.06 to 0.30 mm. The width of the DT8 reaction rim is 0.99 mm. One elongate hole is 3.08 mm long by 0.49–0.86 mm wide and tapers to one side (DT7; Fig. 12D). It has a pronounced reaction rim, with a slightly darkened area directly outside the hole (width  = 0.32 mm) that grades to a thin, dark rim (width  = 0.14 mm). Margin feeding on Lauraceae sp. 2 includes shallow, semicircular feeding (DT12; Fig. 12A) ranging in depth from 0.98–6.42 mm, feeding at the apex through the midvein (DT13; Fig. 12A), and deeper incisions proceeding from the margin toward the midvein (DT15; Fig. 12E), measuring up to 3 mm wide and 5 mm deep. Circular surface feeding marks (DT29; Fig. 12F) with well-developed reaction rims are interspersed with similarly sized holes. Surface feeding marks measure 0.16–0.78 mm in diameter, with a 0.01 mm reaction rim.

A piercing and sucking association on this host plant is characterized by elliptical and ovoidal puncture marks (DT48; Fig. 12G, H). The marks range from 0.25 mm to 0.5 mm in diameter. The centers of the marks are depressed and infilled with a carbonized material. The marks are spread throughout the leaf, but many are produced in close succession, within 1 mm from primary and secondary veins (Fig. 12H).

A host-specific piercing and sucking pattern is found on four specimens of Lauraceae sp. 2 (Fig. 12I–K); circular punctures with central depressions, filled in with dark carbonized material, are arranged in an elliptical or circular pattern (DT281; Appendix 1; Fig. 12J). Marks range in shape from circular to ellipsoidal or comma shaped (Fig. 12K). The individual marks range in diameter from 0.19 to 0.43 mm and tend to increase in size towards the center of the pattern, with the largest marks touching major veins. The marks are mostly positioned around primary or secondary veins, but they are also found on interveinal areas.

Lauraceae species 2 has two associated gall DTs. Circular to ellipsoidal galls with thickened tissue surrounding unthickened inner tissue (DT11, Fig. 12L) are positioned near secondary veins and on interveinal tissue. The galls range in length from 1.66–4.23 mm and in width from 1.16–3.16 mm. The thickened tissue ranges from 0.50–1.50 mm in width. The second gall DT is characterized by dark, circular tissue on interveinal tissue (DT32) and next to secondary veins (DT34; Fig. 12M). There are two groups of four galls arranged together in a quadrangular deployment (Fig. 12M), but most galls are randomly distributed. Their average diameter is 0.41 mm.

### *“Populus” nebrascensis*

**Figure 13:**
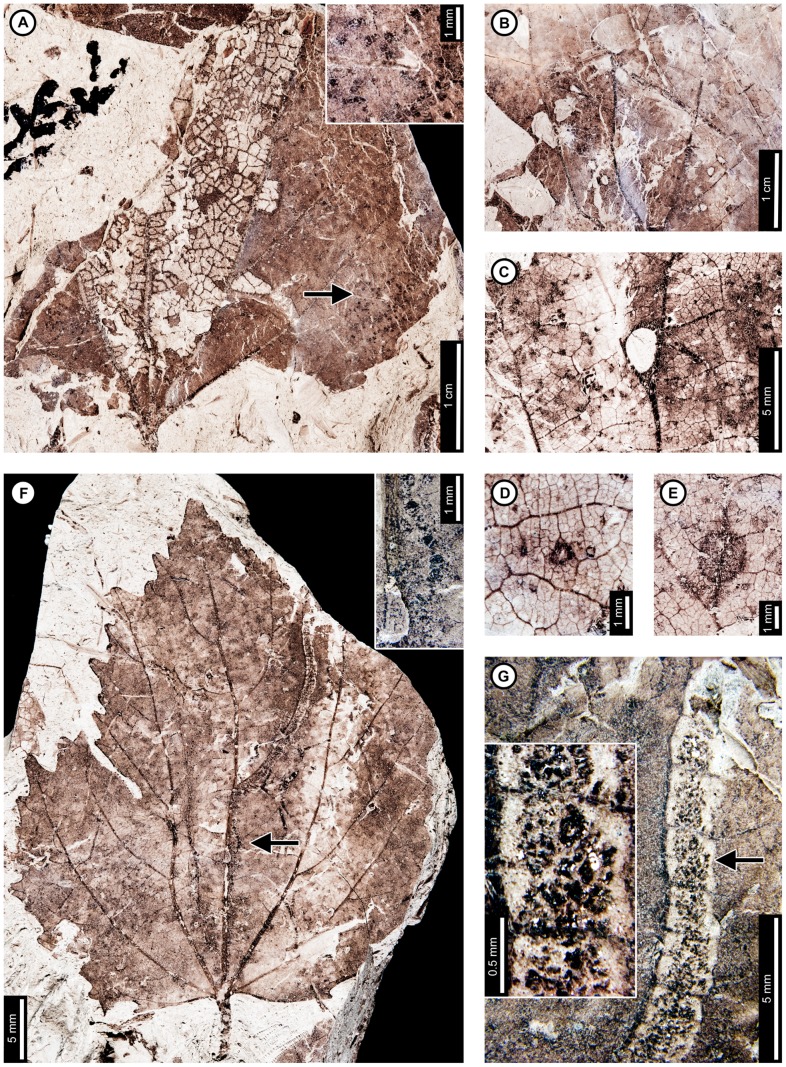
Insect damage on “*Populus*” *nebrascensis*. A: Skeletonized tissue with reaction rim (DT17) bounded by primary veins and possible piercing and sucking marks (DT46). Arrow indicates area expanded in the inset, showing detail of the piercing and sucking marks (USNM 560165). B: Circular holes (DT1) through interveinal tissue and oval holes at the intersection of primary and secondary veins (DT57; USNM 560166). C: Ovoidal hole at the intersection of primary and secondary veins with well-developed reaction rim (DT57; USNM 560167). D: Gall with thickened outer rim and unthickened inner area (DT11) on interveinal tissue (USNM 560167). E. Circular gall positioned on a secondary vein (DT34) with darkened outer rim (USNM 560168). F: Linear mine following the primary vein to a secondary vein, characterized by a gradual width increase and frass-packed trail with spheroidal pellets (DT91). Arrow indicates area expanded in the inset, showing detail of the initial frass trail (USNM 498158). G: Detail of linear path in (F). Closeup of the frass trail and spheroidal pellets in inset. Arrow indicates area expanded in the inset.

Insect damage for “*Populus*” *nebrascensis* is illustrated in Figure 13. External foliage feeding on “*P.*” *nebrascensis* includes hole feeding and skeletonization. Circular holes are 0.68–1.91 mm in diameter, and oval holes are 0.70–5.76 mm long and 0.41–2.80 mm wide (DT1, DT2, DT4; Fig. 13B). Holes are also found at the angular intersections of primary and secondary veins (DT57; Fig. 13B, C) and range from 2.16–4.71 mm in length (measured from the vein intersection to the distalmost hole margin). Reaction rims typically measure 0.18 mm wide. A skeletonized area with reaction tissue (DT17; Fig. 13A) is mostly bounded by primary veins, although small patches cross outside this area. Tertiary venation is preserved, but some tertiary veins were removed during feeding or through taphonomic processes. Small patches of preserved tissue within the skeletonized area are darker than the rest of the unskeletonized leaf, suggesting a reaction to feeding. Reaction tissue surrounding the skeletonized patches outside the primary vein boundaries measures 0.33 mm wide.

Possible circular piercing and sucking marks measure 90 µm in diameter (DT46; Fig. 13A, inset).

“*Populus*” *nebrascensis* has two gall DTs. One probable gall is circular, with dark, thickened tissue surrounding light-colored central tissue (DT11; Fig. 13D). The gall is 0.66 mm in diameter, and the darkened tissue is 0.17 mm wide. There is also a dark, circular gall positioned on a secondary vein (DT34). The gall is 2.38 mm in diameter, and the outer 0.25 mm is darker than the center of the gall (Fig. 13E).

A single mine assigned to DT91 [19] has been found on one specimen of “*P.*” *nebrascensis* (Fig. 13F, G). The mine follows a linear path, initially against the midvein, but it veers off slightly into interveinal tissue before returning to the midvein (Fig. 13F, arrow and inset). The width of the early portion of the mine is 0.33 mm wide and is completely filled with a loosely sinusoidal frass trail. The mine widens to 0.69 mm at the first intersection between the midvein and a secondary vein and follows the secondary vein until termination. At the intersection, the frass trail fills 80% of the mine but is positioned close to the guiding secondary vein. The mine increases in width along the secondary vein, reaching 0.85 mm wide with frass filling the center 60% of the mine. Finally, the mine turns at a tertiary vein and follows the lateral primary vein toward the base until termination. The final 4.5 mm of the mine is free of frass and was probably formed prior to pupation by the last larval instar. Frass pellets are spheroidal, increasing in average diameter from 0.042 mm to 0.077 mm along the secondary vein (Fig. 13G, see inset). The mine does not cross primary or secondary veins, but it does cross tertiary veins. The width of the mine decreases slightly at its intersections with each tertiary vein, giving the margin a wavy appearance (Fig. 13G). Some areas along the margin show darkening, which suggests that the plant reacted to the physical damage caused by feeding.

### *Browniea serrata*

**Figure 14:**
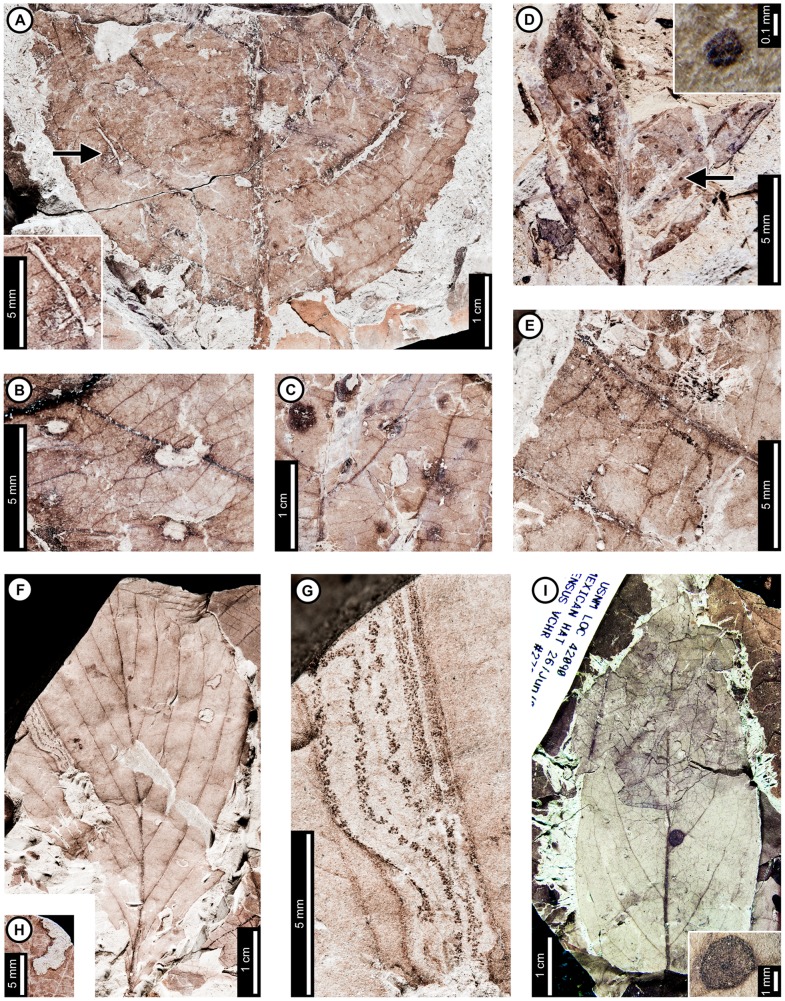
Insect damage on *Browniea serrata* (A–E), “*Ficus*” *artocarpoides* (F–H), and cf. *Ternstroemites aureavallis* (I). A: Circular (DT1, DT2), polylobate (DT3), and elongate slot-feeding (DT8) holes on *Browniea serrata*. Arrow indicates area expanded in the inset, showing detail of the slot-feeding hole (USNM 560169). B: Circular (DT1) and polylobate (DT3) holes on *Browniea serrata*. (USNM 560170). C: Circular galls on interveinal tissue (DT32) and secondary veins (DT34) on *Browniea serrata* (USNM 560171). D: Piercing and sucking marks with central depressions (DT46) on *Browniea serrata*. Arrow indicates area expanded in the inset, showing detail of a piercing and sucking mark (USNM 560172). E. Serpentine mine packed with spheroidal frass pellets (DT91; DMNH 35832) F: Blotch mine with internal frass trail (DT36) and polylobate holes (DT3) on “*Ficus*” *artocarpoides* (YPM 65820). G: Detail of frass trail in (F) showing spheroidal frass pellets. H: Polylobate hole (DT5) on “*Ficus*” *artocarpoides* (USNM 560173). I. cf. *Ternstroemites aureavallis* with a gall at the intersection of primary and secondary veins (DT33). Inset shows details of the gall, including a darkened outer rim and center (USNM 560174).

Insect damage for *Browniea serrata* is illustrated in Figure 14. *Browniea serrata* (formerly *Eucommia serrata* and *Dicotylophyllum anomalum* [49]) features a variety of holes, margin feeding, skeletonization, and galling. Circular holes range from 0.75–2.80 mm in diameter, with darkened reaction rims measuring 0.10 mm wide (DT1, DT2; Fig. 14A, B). All other holes are polylobate (DT3, Fig. 14C), with widths varying from 1.52–2.57 mm and lengths ranging from 2.72–4.17 mm. A 0.10 mm wide reaction rim surrounds the holes (Fig. 14A, B, C). Besides circular and polylobate holes, *B. serrata* also exhibits evidence of slot feeding (DT8; Fig. 14A, arrow and inset). The single slot is rectilinear, parallel sided, and measures 6.4 mm long by 0.44 mm wide. Its reaction rim measures 0.14 mm across. There is one specimen with margin feeding, with a small incision into the leaf margin terminating at a secondary vein (DT15; Fig. 14A). The excision is 1.5 mm wide at the leaf margin and tapers to 0.5 mm wide, meeting a secondary vein at a depth of 3.5 mm. There is a thin reaction rim surrounding the cut area.

*Browniea serrata* is also associated with circular piercing and sucking marks with central depressions, filled with dark carbonized material (Fig. 14D). The marks range in diameter from 0.17 to 0.36 mm. They are spread throughout the leaf, but commonly found near secondary veins.

*Browniea serrata* has one gall association. The galls are darkened circular areas representing thickened tissue and are positioned next to secondary veins (DT34) as well as on interveinal tissue (DT32; Fig. 14C). The average diameters of the galls range between 0.98–1.46 mm.

There is a mine on one specimen of *Browniea serrata*. It is serpentine and filled with what appears to be miniscule, spheroidal frass pellets (DT91; Fig. 14E), although preservation is poor. The preserved mine gradually increases in width from 0.10 to 0.88 mm, but portions of the mine were not recovered. The preserved path of the mine suggests that it crosses secondary veins, probably near the margin of the leaf. There is no reaction tissue along the mine margins.

### *“Ficus” artocarpoides*

Insect damage on “*Ficus*” *artocarpoides* is illustrated in Figure 14F–H, comprising hole feeding and one mine. Holes are polylobate (DT3, DT5), varying in length from 1.95 to 5.54 mm and in width from 0.97 to 2.77 mm (Fig. 14F, H). The mine is a blotch with an internal serpentine frass trail, assigned to DT37 (Fig. 14F, G). The preserved portion is serpentine and sinusoidal. There is a noticeable increase in frass size throughout the trail, increasing from 0.08 to 0.14 mm in diameter. The portion of the frass trail with small frass pellets (∼0.08 mm) is 0.46 mm wide and follows a linear path with continuous frass deposition. The portion with larger pellets (∼0.14 mm) varies in width from 0.18 to 0.40 mm with a curved path and discontinuous frass deposition. The overall width of the mine varies from 2.70–4.26 mm, and secondary veins delimit the widest portions. The mine does not cross secondary veins, and it does not appear to be affected by tertiary venation. The larva may have caused the blotched appearance of the mine by producing an intestiniform feeding path delimited by secondary venation and removing mesophyll tissue between the veins. The margins of the mine are confluent along secondary venation and interveinal tissue. Reaction tissue along the margins of the mine is 0.14–0.18 mm wide.

### cf. *Ternstroemites aureavallis*

One gall was observed on cf. *Ternstroemites aureavallis* (Fig. 14I), positioned at the intersection between the primary vein and a secondary vein (DT33), and crossing the secondary vein. It is circular, with a diameter of 3.24 mm, a darker outer rim measuring 0.75 mm wide, and an inner, circular chamber measuring 0.34 mm in diameter.

### Unknown dicot leaf morphotypes and leaf fragments

**Figure 15:**
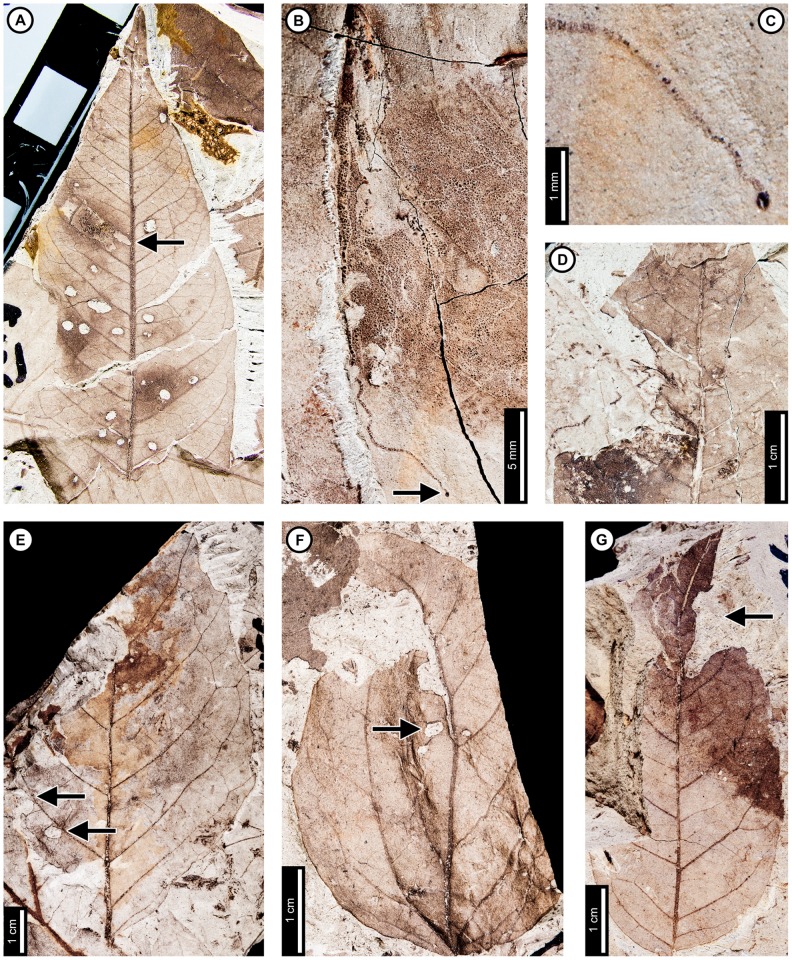
Unknown dicot leaf morphotypes with insect damage. A: Blotch mine delimited by secondary veins with central frass pellets (DT36, arrow), and circular and oval holes (DT2, DT4) on Dicot leaf morphotype 1 (USNM 560178). B: Poorly-preserved serpentine mine on Dicot leaf morphotype 1. Arrow indicates circular oviposition damage (DT91; arrow expanded in C; USNM 560179). C. Detail of (B) showing circular oviposition damage. D: Smooth, semicircular margin feeding (DT12), and probable secondary feeding incision towards the midvein (DT15) on Dicot leaf morphotype 1 (USNM 560180). E: Oval hole feeding (DT2; indicated by lower arrow) and semicircular margin feeding (DT12; indicated by upper arrow) on Dicot leaf morphotype 2 (USNM 560181). F: Oval hole feeding (DT2, arrow) on Dicot leaf morphotype 3 (USNM 560182). G: Deep margin feeding incision reaching the midvein (DT14, arrow) on Dicot leaf morphotype 4 (USNM 560183).

Insect damage for Dicot morphotypes 1–4 is illustrated in Figure 15. The associations on Dicot leaf morphotype 1 (Fig. 15A–D) include hole feeding, margin feeding, and two mine associations. Hole feeding is circular to elliptical, ranging in diameter from 0.16 to 3.52 mm (DT2, DT4) with reaction rims ranging in width from 0.06 to 0.15 mm (Fig. 15A). Smooth, semicircular margin feeding (DT12) measures 1.69 cm across and 0.92 cm deep, with a probable secondary feeding incision towards the midvein measuring 1.47 mm wide and 6.04 mm deep (Fig. 15D). The smooth, large, primary incision resembles leaf-harvesting damage made by extant Megachilidae, the leaf cutting bees [50].

A single blotch mine on Dicot leaf morphotype 1 (Fig. 15A) is bounded by secondary veins and was assigned to DT36 [19]. The left side of the mine is bound by a tertiary vein, and the right side of the mine consists of two polylobate extensions, with the uppermost extension reaching the midvein. There is a dark reaction tissue surrounding the mine, which has a similar appearance in color and shape to the reaction tissue surrounding the hole feeding on the same specimen. The mine is filled with particulate frass. The specimen was not available for further study, and finer details of the frass were not resolvable from the photograph.

Another mining association on Dicot leaf morphotype 1, characterized by a serpentine/linear path, was assigned to DT91 [19] (Fig. 15B). The oviposition site is circular (Fig. 15C) and is followed by an initial threadlike, serpentine trail completely filled with frass and measuring 0.19 mm in width. The second portion of the mine is marked by a moderate width increase to 0.25 mm and a linear path following the leaf margin. The third portion continues to follow the margin, but there is a width increase to 0.52 mm. Frass fills the width of the mine in the first two sections. The center 55% of the last section is filled with frass, and the last 2.81 mm of the mine is free of frass. Because of the poor preservation of the mine, the nature of the frass is indeterminate.

Dicot leaf morphotype 2 (Fig. 15E) has a circular hole (DT2; 15E, lower arrow) measuring 4.40 mm in diameter and semicircular margin feeding (DT12; 15E, upper arrow) measuring 0.70 cm wide at the margin and 0.33 cm deep. Dicot leaf morphotype 3 (Fig. 15F) is associated with oval holes (DT2; Fig. 15F, arrow) that measure 0.12–0.24 cm in diameter. Dicot leaf morphotype 4 (Fig. 15G) features margin feeding assignable to DT14. The removed portion is 1 cm wide at the margin and tapers to 3 mm at the deepest part, where it meets the midvein.

**Figure 16:**
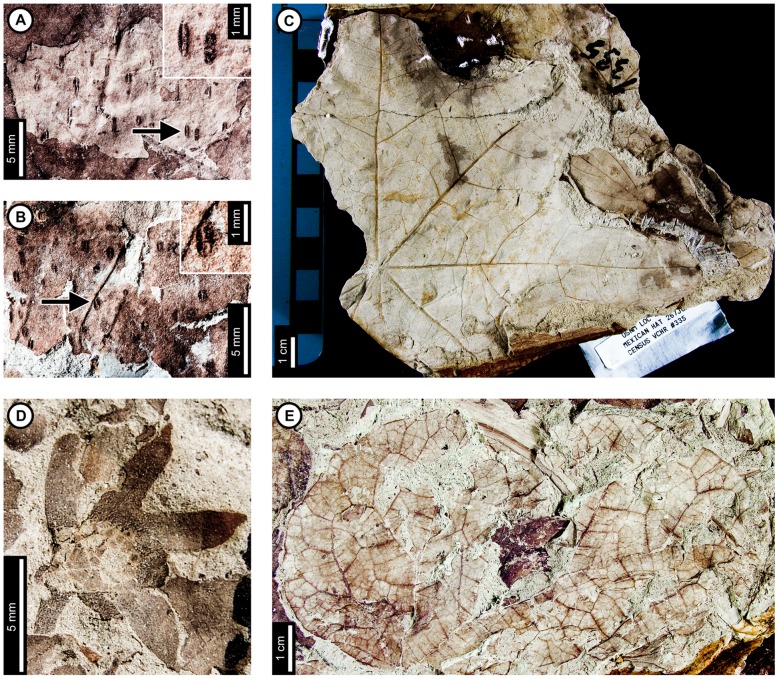
Oviposition (A–B), rare angiosperm leaves (C, E), and one fruit (D) from Mexican Hat. A: Oviposition scars on unidentifiable leaf fragment. Arrow indicates area expanded in the inset, showing detail of oviposition scars (USNM 560184). B: Oviposition scars on unidentifiable leaf fragment. Arrow indicates area expanded in the inset, showing detail of oviposition scar (USNM 560185). C. *Paleonelumbo macroloba* leaf, without insect damage (rotated to fit layout; USNM 560175). D: *Polyptera manningii* fruit, which is correlated with *Juglandiphyllites glabra*, without insect damage (USNM 560176). E: *Paranymphaea crassifolia* without insect damage (USNM 560177).

Oviposition scars are found on three unidentifiable leaf fragments (Fig. 16A–B). The scars are ovoid or elliptical and measure between 0.45–1.70 mm in length and 0.40–0.70 mm in width. The thickened reaction tissues surrounding the entry slits measure 0.15–0.30 mm wide. Many oviposition scars appear on each damaged leaf but are not arranged in any discernable pattern.

### Leaves and fruits without insect damage

Undamaged leaves and fruits are illustrated in Figure 16C–E. Insect damage was not found on *Paleonelumbo macroloba* (Fig. 16C) and *Paranymphaea crassifolia* (Fig. 16E) leaves nor on *Polyptera manningii* fruits (Fig. 16D) at Mexican Hat. The lack of damage can probably be explained by the small sample size examined for each species, namely two specimens each of *Paleonelumbo macroloba* and *Paranymphaea crassifolia*, and one specimen of *Polyptera manningii*. Leafy branches of the only conifer, *Glyptostrobus europaeus* (Cupressaceae) were very abundant; although they were not censused, damage on this species was noted as extremely rare (PW and CCL pers. obs. 2004).

## Results and Discussion

Our results indicate both that leaf-mining and presumed leaf-miner diversity at Mexican Hat is even higher than previously recognized, and, equally importantly, that none of the Mexican Hat mines can be linked back to the local Cretaceous mining fauna. Thus, there is no evidence for survivorship of any of the diverse Cretaceous leaf miners over the K-Pg boundary, even on well-sampled, surviving plant families (Cercidiphyllaceae and Platanaceae). These results show that the high insect damage diversity at Mexican Hat represents an influx of novel insect herbivores during the early Paleocene and not a refugium for Cretaceous leaf miners.

### Affinities of Mexican Hat mines

Lepidopterans are the most diverse leaf-mining insects [51]. The leaf-mining habit is practiced by basal lepidopteran groups and may have only evolved once [52], [53]. Numerous probable lepidopteran mines have been described in the fossil record [54], dating back to the Early Cretaceous [55] or possibly latest Jurassic [56], [57], although the lepidopteran leaf-mining habit may extend considerably earlier, to the mid Jurassic [58]. The abundance of fossil lepidopteran mines, compared to other orders, may be attributable to the early origin of mining behavior within Lepidoptera or to their overall preference for woody over herbaceous plants [53], [59], the latter of which are much less likely to be fossilized.

Lepidopteran miners make serpentine and blotch mines, and mine paths rarely overlap with earlier traces. Most produce solid frass deposited in pellets. The frass trails are usually deposited in a medial, linear path, or dispersed throughout the mine width [41]. Exit holes are small and circular [41], semicircular [42], or in slits [60]. These characteristics, especially mine shape and frass, suggest that lepidopteran larvae made most of the mines at the examined localities. Mexican Hat mines included in DT37, DT41, DT91, and DT92, all exhibit features associated with lepidopteran mines, and they share many similarities with those of extant Nepticulidae and Gracillariidae micromoths. Nepticulidae larvae create serpentine or linear mines with a continuous central frass trail, often composed of ellipsoidal or spheroidal frass pellets. The widths of the mines increase with each instar [61]. Gracillariidae larvae typically make significantly larger blotch and serpentine mines. Their serpentine mines, often produced by species of *Phyllocnistis*, are characterized by a median frass trail, which typically extends unbroken through the mine, except for the last instar immediately before creation of the terminal chamber. The final instar does not feed, and it makes a cocoon in the mine terminus [60]. The mines on *J. glabra* that lack evidence of frass (DT42 and DT105; Fig. 6A, 4I), unless this is taphonomic, were probably also made by lepidopteran larvae because almost all miners known to remove frass from their mines are Lepidoptera [41]. Alternatively, these mines could have been made by a sap-feeding species.

Leaf-mining Hymenoptera occur within one of the basal sawfly superfamilies, Tenthredinoidea [59], [62], particularly in the families Tenthredinidae, Argidae, and Pergidae [59]. Most hymenopteran leaf miners are found in Tenthredinidae subfamilies Heterarthrinae and Nematinae, which evolved mining behavior separately from each other [63]. The fossil record of Tenthredinoidea is fairly rich and extends back to the Jurassic [64]. The earliest putative tenthredinid is from the earliest Cretaceous, but this record may represent a stem group [65], [66]. Many fossil sawflies from the Eocene have been assigned to Tenthredinidae [67]–[72].

Tenthredinidae generally produce blotch mines with pelletized frass that is dispersed irregularly through the mine or near the margins. The mine may begin with a short serpentine phase or start as a blotch. Exit holes are typically large and circular [41]. This Tenthredinidae mine morphology matches the DT36 mines on *P. raynoldsii* (Fig. 3A–D). There is one known species of Tenthredinidae associated with extant *Platanus* (Platanaceae), *Bidigitus platani*, which mines *Platanus racemosa* at least in the Californian part of its range [73]–[75]. The mine morphology of this modern association differs from DT36 on *P. raynoldsii* because the *B. platani* mine has a short, initial serpentine phase. The presence of Tenthredinidae mines at Mexican Hat on *Platanus* extends the Tenthredinidae-*Platanus* mining association to the early Paleocene. Other possible fossil Tenthredinidae leaf mines have been described on host plants other than *Platanus*, including from the Miocene of Romania [76], and others have noted morphological similarities between extant Tenthredinidae mines and leaf mine fossils from the Albian and Turonian [77], [78] and the Eocene [79].

Dipteran leaf miners are found in several basal and derived fly families, but the most speciose is Agromyzidae [80]. Agromyzids produce serpentine and blotch mines, often with irregular paths and overlapping sections. They do not feed on cell walls, are limited to sap-feeding by the mouthhook design of their mouthparts, and produce fluidized frass, which forms small clumps. Because the larvae (maggots) feed on their sides relative to the leaf surface and often alternate between borders of the mine, their frass may be deposited in alternating rows. A thorough description of dipteran mine characteristics, particularly of Agromyzidae, was given by Winkler et al. [26]. The agromyzid mine on *P. raynoldsii* at Mexican Hat, *Phytomyzites biliapchaensis*, represents an extinct association because no modern agromyzids feed on *Platanus*. This contrasts with the proposed Tenthredinidae mine (DT36) on *P. raynoldsii*, which appears to represent a long-term surviving relationship between Tenthredinidae and *Platanus*.

Coleoptera may have been the first order of leaf miners, possibly responsible for the earliest known mines, from the Triassic (i.e., [81], [82]). Coleopteran mines can resemble lepidopteran mines, although coleopteran excision areas are usually larger and typically form robust, full-depth mines with particulate frass [41]. Leaf-mining coleopterans are much less diverse today than leaf-mining lepidopterans. No clear evidence of coleopteran mines has been found at Mexican Hat.

### Mining diversity and host specificity at Mexican Hat

Leaf mine diversity at Mexican Hat is much higher than at any other Paleocene locality examined in this study, or at any other Paleocene site from the United States known to the authors, who have collectively excavated at least 200 Paleocene plant localities from the Rocky Mountains and Great Plains. The full suite includes 9 mine DTs on 8 host plants (18 insect-host plant pairs total; Table 3), increasing from an earlier count of 7 mines on 6 host plants (14 DT-host plant pairs total) [19]. However, as detailed below, host-specific variation strongly suggests that an even higher number of actual mining species was present at Mexican Hat. For comparison, Pyramid Butte, which lies just 20 cm above the K-Pg boundary 15 km north of Marmarth, North Dakota, had the next highest number of mine DTs (2) out of the early Paleocene sites. Mines at Pyramid Butte were found on 2 host plants (2 insect-host pairs total) from a collection of 549 specimens. The Battleship locality, located 3.6 m below the K-Pg boundary 5 km northeast of Marmarth, had the highest number of mines at a Cretaceous site, 5 mine DTs on 10 hosts (10 insect-host plant pairs total), although somewhat less than half the number of specimens were sampled (459) than at Mexican Hat (1073 specimens). When compared by resampling at equal sample size (400 leaves) [19], Battleship had ca. 5 mine DTs and Mexican Hat ca. 3 mine DTs.

**Table 3:** Mine damage types by host plant at Mexican Hat.

Host Plant	Leaves	Mine DTs	Leaves with Mines
*Platanus raynoldsii*	1207	DT36	3
		DT91	20
		DT104	63
		DT282	1
*Juglandiphyllites glabra*	396	DT42	1
		DT91	4
		DT92	2
		DT105	1
		DT282	1
*Zizyphoides flabella*	231	DT41	1
		DT91	6
*Cercidiphyllum genetrix*	214	DT41	1
		DT91	1
“*Populus*” *nebrascensis*	85	DT91	1
*Browniea serrata*	12	DT91	1
“*Ficus*” *artocarpoides*	4	DT37	1
Dicot morphotype 1	12	DT36	1
		DT91	1

Leaf-mining larvae produce characteristic damage that can provide taxonomic clues [41], but many species produce very similar morphologies. Four mine DTs at Mexican Hat appear on multiple host plants, but their placement within a single DT in no way implies that all the damage was made by the same insect species. Rather, because most leaf miners are specialized [53], mine characters unique within one host plant may suggest one-to-one host specificity of the leaf-mining insect species. Mine DTs occurring on multiple host plants include DT36 (*P. raynoldsii*, Dicot leaf morphotype 1), DT41 (*C. genetrix*, *Z. flabella*), DT91 (*P. raynoldsii*, *Z. flabella*, *J. glabra*, *C. genetrix*, “*P.*” *nebrascensis*, *B. serrata*, Dicot morphotype 1), and DT282 (*P*. *raynoldsii* and *J*. *glabra*). The following analysis of fine differences within DTs, among host plants, suggests a higher number of leaf-mining species at Mexican Hat than implied by the number of mine DTs.

Blotch mines assigned to DT36 are associated with five *P. raynoldsii* specimens (Fig. 3A–D) and one Dicot morphotype 1 specimen (Fig. 15A). Damage type 36 mines on both hosts exhibit some similarity, including lack of a central chamber, frass pellets dispersed throughout the mine, and channeling of the mine path by secondary venation. However, host-specific differences also are present. The mines on *P. raynoldsii* contain initially small and closely packed frass. Larger pellets, produced by later instars, are spread farther apart (Fig. 3D). The differently-sized frass pellets do not usually overlap, allowing for the inference of larval instar shifts and feeding trajectory. In contrast, frass in the DT36 mine on Dicot morphotype 1 is packed closely together near the center of the mine, with no clear spatial differentiation in pellet sizes (Fig. 15A; a more detailed look at the frass was not possible because the specimen was unavailable). Although the mine on Dicot morphotype 1 does not cross secondary veins for an extensive range, parts of the secondary vein were removed where the larva crossed the vein. Removal of vein tissue also is observed along the primary vein (Fig. 15A). Vein tissue removal is not evident on any of the DT36 mines on *P. raynoldsii*. The DT36 mine on Dicot leaf morphotype 1 has a thick reaction rim surrounding the mine margin (Fig. 15A), but reaction tissue is not apparent on the *P. raynoldsii* mines. The overall sizes of the DT36 mines on *P. raynoldsii* are larger. In summary, it is very likely that these occurrences of DT36 on two different host plants represent separate leaf-mining taxa.

Mines of the DT41 type are associated with one specimen each of *Z. flabella* (Fig. 8F–H) and *C. genetrix* (Fig. 10E–G). The mines on both specimens exhibit serpentine paths with expanding widths and smooth margins, and although affected by secondary venation, other host-specific differences also are apparent. The DT41 mine on *Z. flabella* begins with an extended threadlike phase measuring around 0.10 mm wide (Fig. 8H), compared to a 0.27 mm wide initial phase on *C. genetrix* (Fig. 10G). Frass fills the width of the mine on *Z. flabella* (Fig. 8H), but it fills only 32–65% of the center of the mine path on *C. genetrix* (Fig. 10F) The mine on *Z. flabella* also features a dramatic widening associated with loosening of the otherwise tightly packed frass trail (Fig. 8G), whereas no evidence of a change in frass trail density is noticeable on *C. genetrix*. The overall length of the mine on *Z. flabella* is also much greater, including a longer threadlike phase, compared to the mine on *C. genetrix*, although the latter specimen is not fully preserved. In summary, it is very likely that these two instances of DT41 on different host plants represent separate leaf-mining taxa.

Mines assigned to DT91 are associated with 20 specimens of *P. raynoldsii* (Fig. 2A–H), 6 specimens of *Z. flabella* (Fig. 8A–E), 4 specimens of *J. glabra* (Fig. 5A–D), 1 specimen of *C. genetrix* (Fig. 10A–D), 1 specimen of “*P.*” *nebrascensis* (Fig. 13F–G), 1 specimen of *B*. *serrata* (Fig. 14E), and 1 specimen of Dicot morphotype 1 (Fig. 15B–C). Similarities among all DT91 mines at Mexican Hat include a serpentine mine path, ellipsoidal frass pellets, pronounced width expansion, and control of mine margins by tertiary venation.

Despite these similarities, several host-specific differences exist among DT91 mines (Table 4). One distinct feature of DT91 mines on *P. raynoldsii* is a dramatic increase in width at the terminal portion (Fig. 2A, D, E, G). Secondary veins typically delimit the mine path, leading to a winding pattern (Fig. 2A, E). On other specimens, the mine continues on a similar path, but the width of the mine greatly increases, and frass pellets are deposited in clusters near the margin (Fig. 2D, G). The larvae that produced DT91 mines on *P. raynoldsii* fed through tertiary venation during the terminal phase of the mine, a syndrome not observed on any other host plant (Fig. 2A, E). The DT91 mines on *Z. flabella* originate near a secondary vein and loop around to the adjacent secondary vein, following it until termination (Fig. 8A, D, E). The single DT91 mine on *C. genetrix* follows a similar trajectory to that on *Z. flabella*, but the frass trail ends 22 mm before the terminus. A frass-free terminus is common in DT91 mines, but the length of the frass-free phase on *C. genetrix* is much longer than on any other observed specimen (Fig. 10B). The frass trail on *C. genetrix* is also unique for DT91 at Mexican Hat, with spheroidal frass pellets embedded in a non-particulate frass matrix (Fig. 10C–D). The frass trail of DT91 mines on *J. glabra* also differs from mines on other host plants. Frass is spheroidal and spread out in a loose trail or completely absent in some areas (Fig. 5A–D). The distance between frass pellets is greater than for similar mines on other host plants. The DT91 mine on “*P.*” *nebrascensis* follows a linear path along primary and secondary veins through its entire course, which has not been observed on any other DT91 mine (Fig. 13F–G). The DT91 mine on Dicot morphotype 1 follows a linear path along the margin initially, but then turns along a secondary vein and widens (Fig. 15B). A circular oviposition site is clearly visible at the origination of the mine (Fig. 15C) but is not observed on any other host plants. Individual frass pellets are ambiguous, but this may be an artifact of preservation. The DT91 mine on *B. serrata* follows a serpentine path across secondary veins. The frass trail, possibly made up of spheroidal frass pellets, appears to fill the entire mine width, although preservation is poor, and the specimen is fragmented.

**Table 4:** Comparison of DT91 leaf mine morphology by host plant at Mexican Hat.

Plant host	Mine width (mm)	Margins	Frass trail percentage	Pellet shape	Pellet length x width (µm)	Distinctive features	*#*
*P. raynoldsii*	0.32–7.89	wavy	41–77%	spheroidal/ellipsoidal	15–130×10–75	dramatic widening during terminal phase, removal of tertiary veins	20
*J. glabra*	0.38–2.33	wavy	25–70%	spheroidal	50×50	distinctive spheroidal frass, sparse/spread out frass trail	4
*Z. flabella*	0.45–1.9	wavy	30–50%	ellipsoidal	45–90×45–82	close association with secondary veins, most densely packed frass trails of DT91 mines	6
*C. genetrix*	0.28–0.90	initially smooth, then wavy	62–43%	spheroidal with fluidized frass matrix	33×33	frass trail composed of spheroidal pellets in a fluidized frass matrix	1
“*P.*” *nebrascensis*	0.33–0.85	wavy	100–80%	spheroidal/ellipsoidal	42–77	linear path along primary to secondary vein	1
*B. serrata*	0.10–0.88	smooth	100%	probably spheroidal	N/A	N/A – poor preservation and incomplete fossil	1
Dicot morphotype 1	0.19–0.52	smooth	100–55%	unknown	N/A	oviposition damage mark at proximal trail	1

Frass trail percentage range from earliest trail to latest trail.

Differences in DT91 mines among host plants strongly suggest that more than one species made them. Evidence for multiple leaf-mining species is clearly seen, for example, by comparing the dramatic width increase and winding path on *P. raynoldsii*, the loosely packed frass trail with spheroidal pellets on *J. glabra*, the sparse frass pellets surrounded by a possibly fluidized frass matrix on *C. genetrix*, and the oviposition site damage on Dicot leaf morphotype 1. These differences allow for a conservative estimate of five leaf-mining species, although seven or more are possible.

Epidermal mines assigned to DT282 occur on *P*. *raynoldsii* (Fig. 3E) and *J*. *glabra* (Fig. 4K) at Mexican Hat. A serpentine path with dark frass or reaction tissue along the margins and a frass-free central chamber characterize this DT. Mines on both host plants feature similar morphologies, but both specimens are poorly preserved and lack proximal and terminal portions. The lack of preserved features prevents comparisons of the full range of mine characteristics. Based on shared distinctive morphological traits, however, DT282 mines on *P*. *raynoldsii* and *J*. *glabra* were probably made by closely related species.

Despite the fine variation within mine DTs among host plants detailed above, there are potential issues with precisely inferring species-level mining diversity based on minor host-specific differences among leaf mines. Leaf architecture varies among host plants and can affect mine paths. Mining larvae may avoid or follow leaf veins because of tissue-specific variations in nutritional quality. Vein arrangement may be one of the most important factors controlling feeding path and mine shape [83]. Because leaf anatomical characters differ among plant species and affect mine path, care must be taken to avoid interpreting slight differences in path morphology driven by venation differences as representing different mining species. Seasonal changes can affect leaf nutritional quality, and in turn affect the amount of time spent feeding within a leaf and overall mine length [41]. Nutritional differences among host plant species may also have an effect on mine length for polyphagous leaf miners. However, despite possible issues with interpreting slight host-specific variations among mines, we have listed above many host-consistent differences that are not obviously related to leaf venation or other factors.

In conclusion, the species-level diversity of mining insects at Mexican Hat must have been significantly higher than the number of DTs originally assigned to those mines [18]. Six mine DTs were recorded in [19], and two additional mine DTs were added in this study, but host-specific morphological differences among mine DTs, detailed above, suggest higher mining diversity than previously recognized. In summary, these results suggest that at least 15 insect species created the leaf mines at Mexican Hat, but the presence of 18 or perhaps even more mining species is possible.

### No survivors? Comparison with Cretaceous leaf mines

We found no conclusive evidence for the survival of any of the diverse Cretaceous leaf-mining fauna over the K-Pg boundary (Table 5). Earlier studies [11], [19] found four mine DTs that crossed the boundary in the Western Interior, USA: DT41, DT42, DT43, and DT59. Two of these (DT41 and DT42) occur at Mexican Hat. In the case of DT43 (not illustrated), the differences between Hell Creek and Fort Union samples were so great on re-examination that we did not consider this an example of a possible surviving leaf mine association. Damage type 59 has not been found at Mexican Hat, but it has been found in the supplemental local Paleocene collections and therefore is discussed here. As detailed below, although there are some morphological similarities within the DTs that cross the boundary, there is no convincing evidence to suggest that the damage was made by the same leaf-mining taxon. The potential boundary-crossing DTs are also not found on related host plants.

**Table 5:** Comparison of leaf mine damage types from the Cretaceous Hell Creek Formation, Williston Basin, North Dakota; local early Paleocene localities in the Fort Union Formation, Powder River and Williston Basins, of North Dakota and Montana; and Mexican Hat, Powder River Basin, Montana.

Mine DT	Hell Creek Fm.	Mexican Hat	Local Early Paleocene
DT35	x		
DT36		x	
DT37	x		
DT40	x		
DT41	x	x	
DT42	x	x	
DT43	x		x
DT45	x		
DT59	x		x
DT60			x
DT65	x		
DT90		x	
DT91		x	x
DT92		x	
DT104		x	
DT105		x	
DT109	x		
DT282		x	x

**Figure 17:**
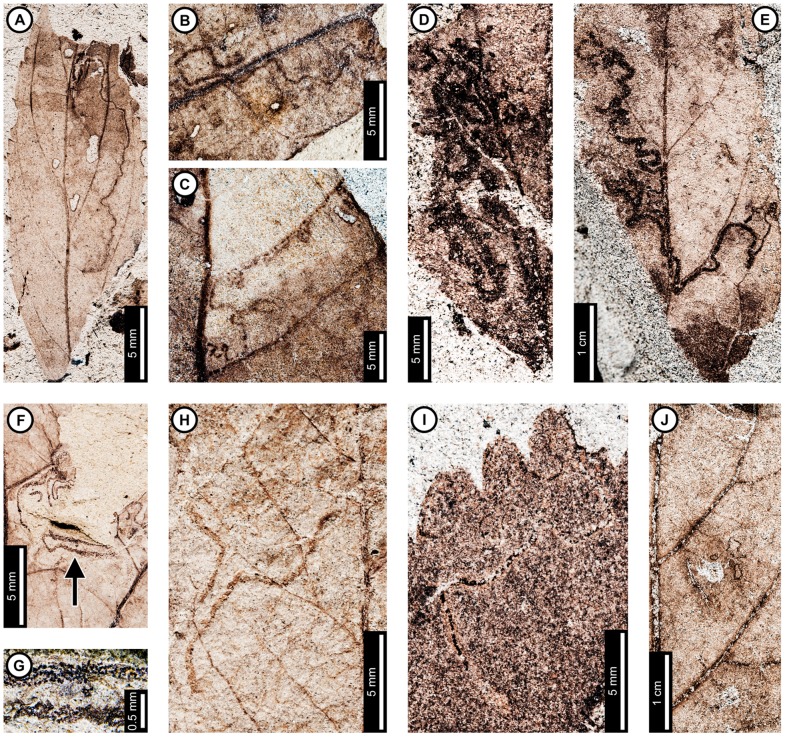
Mines assigned to DT41 from the latest Cretaceous of North Dakota and Montana, USA. A: Thin, serpentine mine, characterized by a gradual width increase and central frass trail (HC81; Somebody's Garden – DMNH loc. 9824; DMNH 19619). B: Frass-packed serpentine mine with minimal width increase and angular turns on “*Artocarpus*” *lessigiana* (Triceps – DMNH loc. 1855; DMNH 20005). C: Threadlike mine with looping path (HC34; Allison's Attachment – DMNH loc. 568; DMNH 20503). D: Leaf mine with tightly coiled, intestiniform path, packed with frass (Mud Butte, Dean Street – DMNH loc. 428; DMNH 7483). E: Serpentine leaf mine packed with frass (Unidentifiable; Triceps – DMNH loc. 1855; DMNH 7511). F: Partially preserved serpentine leaf mine with gradual width increase and dispersed, spheroidal frass pellets (arrow expands to G; Unidentifiable; Triceps – DMNH loc. 1855; DMNH 20000). G: Detail of frass trail in (F) showing dispersed, spheroidal frass pellets. H: Smooth-margined, linear mine with parallel sides on “*Dryophyllum*” *subfalcatum* (Dragonfly – DMNH loc. 565; DMNH 7908). I: Serpentine mine with thin, central frass trail starting near leaf apex (Skunk Hunt – DMNH loc. 4301; DMNH 35834). J: Aborted mine (HC199 (Laurales); Mud Butte, Dean Street – DMNH loc. 428; DMNH 7544).

Damage type 41 (Fig. 17) is one of the most common mine DTs from the Cretaceous North Dakota sites, with 22 specimens censused in [11] and 6 additional specimens examined in this study. The earliest local (Williston Basin of North Dakota) appearance of this mine DT is 103 meters below the K-Pg boundary, and the latest known local Cretaceous appearance is 3.6 m below the K-Pg boundary at the Battleship locality [10], [11]. Paleocene DT41 mines are found at Mexican Hat and at several late Paleocene sites in Wyoming. Although similar morphological characteristics, such as a serpentine path with smooth margins, are observed consistently in DT41 mines from both the Cretaceous and Paleocene, there are many host-specific morphological differences that suggest that DT41 mines in the Cretaceous were made by numerous different leaf-mining species, whereas the Paleocene DT41 mines were made by only a few species.

A consistent feature of all Paleocene DT41 mines found in this study is the position at the base of a multi-veined leaf, occurring on *Z. flabella* (Fig. 8F) and *C. genetrix* specimens (Fig. 10E–K). No examined Cretaceous DT41 mine is situated in a similar position (Fig. 17). The consistently small size (length and width) of some Cretaceous leaf mines precludes them from being related to any known Paleocene DT41. For example, a delicate mine associated with leaf morphotype HC81 (Fig. 17A) is initially around 0.13 mm wide and filled entirely with frass. The mine widens to 0.45 mm by its terminus, and only the middle 35% of the mine is filled with frass. The widest portion of the mine has less than half the greatest width of any Paleocene specimens. Another Cretaceous mine, on “*Artocarpus*” *lessigiana* (Fig. 17B), increases minimally in width throughout its path, from 0.25–0.30. The mine path also makes angular turns, which differs from observed Paleocene mines. Other Cretaceous mines assigned to DT41 differ in path morphology. Examples include a tightly sinusoidal and looping path on leaf morphotye HC34 (Fig. 17C), a coiled and intestiniform path (Fig. 17D), a tightly sinusoidal path with many angular turns on an unidentified leaf (Fig.17E), and a mostly linear mine lacking any major width increase on “*Dryophyllum*” *subfalcatum* (Fig. 17H). Differences in frass trail morphologies also occur in Cretaceous DT41 mines compared to examined Paleocene specimens. One example occurs on an unidentified leaf (Fig. 17F) and on the leaf morphotype HC266 (DMNH 20015). The mines are initially threadlike but widen throughout their course. The full widths of the mines are filled with spheroidal frass pellets that increase in size and spread farther apart as the mine widens (Fig. 17G). Aborted mines (Fig. 17J) have also been included in DT41. These mines are typically around 0.1 mm in width with no width increase, and 5 mm or less in length. Other Cretaceous DT41 mines have a frass-free terminal portion, measuring ca. 2.8 mm in length (Fig. 17I). All examined Paleocene DT41 mines are filled with frass until termination.

The consistent positioning of mines at the bases of multi-veined leaves and association with basal primary veins, as found on Paleocene DT41 on *C. genetrix*, has not been found on any examined Cretaceous specimens. These characteristics, plus the extended threadlike phase followed by an expansion into a loosened frass trail observed on *Z. flabella* at Mexican Hat, further suggest that leaf miners responsible for DT41 mines at Mexican Hat and in the local Paleocene differed from those of the Cretaceous.

Another potential K-Pg boundary crosser, DT42, (Fig. 6), was found in the latest Cretaceous of North Dakota (five specimens, Fig. 6B–C) [11] and Mexican Hat (one specimen, Fig. 6A). Mines assigned to DT42 are defined by a linear, widening path, which lacks frass, and ragged margins. All Cretaceous DT42 specimens differ from the Mexican Hat specimen (Fig. 6A). A typical Cretaceous DT42 (Fig. 6B) features a linear skeletonized portion that follows a secondary vein before a dramatic expansion to a small blotch with thickened reaction tissue. Another Cretaceous mine (Fig. 6C) follows primary and secondary veins. The mine expands dramatically towards the end of its path, starting at 0.61 mm and widening to 1.04 mm, although it is possible that this is an incomplete mine. The 41% width expansion is about half of the expansion of the Mexican Hat specimen (81%). Tissue at the end of the Cretaceous mine was completely removed. The Mexican Hat DT42 (Fig. 6A) expands in width throughout the preserved portion and is strongly influenced by primary and secondary venation. Part of the margin is wavy because it is delimited by tertiary venation. The gradual expansion differs from most Cretaceous specimens, which exhibit a dramatic widening near termination (Fig. 6B, C). In summary, it is unlikely that the Cretaceous and Paleocene DT42s represent the same taxa.

**Figure 18:**
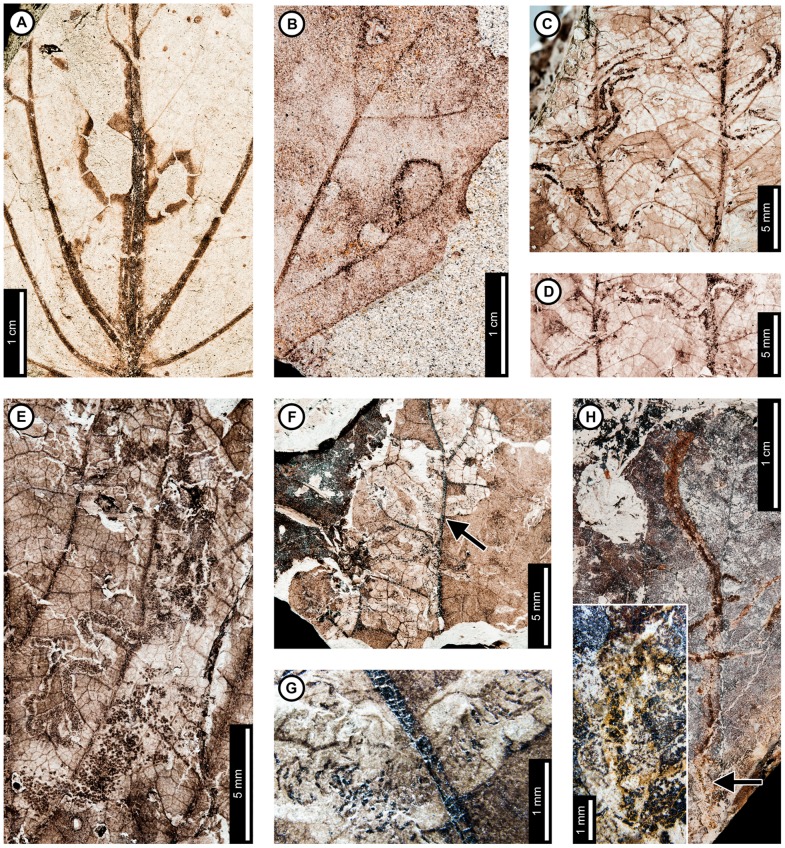
Leaf mines assigned to DT59 (A–B), DT282 (C–D), and DT91 (E–H). A: Two linear leaf mines with widened terminal chambers along the midvein of *Paranymphaea crassifolia* (DT59; Paleocene; Paleocene Leaf –DMNH loc. 563; DMNH 20055). B: Leaf mine with widened terminal chamber along secondary vein on leaf morphotype HC265 (DT59; Cretaceous; Battleship – DMNH loc. 900; DMNH 7286). C, D: Probable mines on “*P.*” *nebrascensis* from Pyramid Butte, ND (DT282; YPM locality 86107; YPM 9796, YPM 9762). E. Initially serpentine mine with blotch-like terminal chamber on “*P.*” *nebrascensis* (DT91; YPM loc. 87150; YPM 9636) F. Serpentine mine with loosely packed frass trail on an unidentifiable leaf fragment (DT91; arrow expanded in G; Pyramid Butte - YPM loc. 86107; YPM 168194) G. Detail of frass trail in (F) showing meniscate pattern. H. Serpentine mine with medial frass trail composed of spheroidal-ellipsoidal pellets on *Z*. *flabella* (DT91). Arrow indicates area expanded in the inset, showing detail of the frass trail (YPM loc. 8403; YPM 9526).

Damage type 59 mines are characterized by a linear path adjoining and following secondary and primary veins. The mine path increases minimally in width, but it ends with an expanded ovoidal or ellipsoidal terminal chamber (Fig. 18A). The two Cretaceous DT59 mines are much smaller than both mines on the Paleocene specimen (which is from the site Paleocene Leaf, DMNH loc. 563, and not Mexican Hat; Fig. 18A). The Cretaceous DT59 in Fig. 18B only follows a secondary vein before expanding into an ovoidal terminal chamber measuring 5.07×7.14 mm. The mine path increases from 0.39 mm wide at the oviposition site to 0.81 mm wide surrounding the terminal chamber. The Paleocene DT59 path is 0.56 mm wide and expands to 1.98 mm wide surrounding the terminal chamber, which measures 7.12×11.83 mm (Fig. 18A). The overall sizes of the termini of the Paleocene DT59 specimens are much larger than on the Cretaceous specimens, and the association with both secondary and primary veins is not observed in either Cretaceous specimen.

The variety of morphologies within Cretaceous DT41 mines and other boundary crossing DTs, and the number and identities of hosts that they are found on, suggest that the diversity of leaf-mining Lepidoptera was significantly greater during the latest Cretaceous than previously recognized. Similarly, the lack of evidence for the survival of any leaf-mining forms over the K-Pg boundary suggests a near total extinction of leaf miners [11]. In contrast, the analysis of Paleocene DT41 mines in this study did not suggest greater diversity than recognized in past studies [19]. With similar collection sizes, DT41 mines were found on over 20 Cretaceous host plants and only 3 host plants in the Paleocene, further suggesting a major extinction of the local microlepidopteran mining fauna.

### Comparison of Mexican Hat and other Paleocene insect damage

Three Mexican Hat leaf mine DTs (DT41, DT91, and DT282) are found at other local or regional Paleocene sites. Damage type 41 is associated with *Z. flabella* (Fig. 8F–H) and *C. genetrix* at Mexican Hat (Fig. 10E–G) and with *C. genetrix* at the early late Paleocene (ca. 59 Ma) sites Haz-Mat, Washakie Basin of southwestern Wyoming (Fig. 10H–I), and Skeleton Coast, Bighorn Basin of northwestern Wyoming (Fig. 10J–K). All DT41 mines on Paleocene *C. genetrix* arise at the base of the multi-veined leaf and are usually associated with primary veins. A serpentine path, packed with frass and increasing in width throughout, defines the mines. Mine width is initially around 0.1 mm and widens up to 0.95 mm, and margins are typically smooth.

The presence of the *C. genetrix*-DT41 association at Mexican Hat, Haz-Mat, and Skeleton Coast shows that this mining association persisted for at least 6 million years, thus providing a striking but singular example of a long-lasting leaf-mining association from Mexican Hat. Earlier studies have also provided evidence for the persistence of insect-plant relationships over geologic time. Opler [44] suggested a minimum Miocene age for eleven leaf mine associations based on the indistinguishable morphology of extant and fossil mines on related oaks. Hispine beetle surface-feeding damage has been found on Zingiberales in the latest Cretaceous (Hell Creek Formation) and regional Eocene, and the association still occurs today in the Neotropics [84]. Biogeographic evidence has also been used to suggest long-term insect-plant relationships and to explain modern distributional patterns [85], [86]. Fossil evidence for long-term associations between plants and insects has usually been found at the family or genus level, but here we present a one to one insect-plant association spanning 6 million years that may have involved a single, or perhaps a few closely related mining species.

Probable mines, assigned to DT282 and associated with *P. raynoldsii* (Fig. 3E) and *J. glabra* (Fig. 4K) at Mexican Hat, are also found on three “*P.*” *nebrascensis* specimens (Fig. 18C–D) at Pyramid Butte, ND. The morphology is very similar among mines on all three hosts, although the proximal and terminal portions are not preserved on any of the specimens, suggesting a taphonomic bias against preservation of these features. Major veins do not affect the mine paths, suggesting that the mines traveled through epidermal tissue. The mines are all poorly preserved, which prevents detailed comparison of the frass. Despite the poor preservation, the mines are morphologically similar overall, which suggests that they were made by a few species of closely related epidermal miners.

Damage type 91 mines are associated with *P*. *raynoldsii*, *J*. *glabra*, *Z*. *flabella, C*. *genetrix*, “*P.*” *nebrascensis*, *B*. *serrata*, and Dicot leaf morphotype 1 at Mexican Hat, and they are also found at three other early Paleocene localities (“*P.*” *nebrascensis* at YPM loc. 87150, ND; *Z. flabella* at YPM loc. 8403, ND; unidentifiable leaf fragment at Pyramid Butte, ND). The mine on “*P.*” *nebrascensis* at YPM loc. 87150 (Fig. 18E) is initially serpentine, with a densely-packed, sinusoidal frass trail composed of spheroidal-ellipsoidal pellets. The terminal portion widens dramatically into a blotch-like mine filled with ellipsoidal frass pellets. This morphology is most similar to DT91 mines on *P*. *raynoldsii* at Mexican Hat (Fig. 2), which typically increase dramatically before termination. The mine on the unidentifiable leaf fragment from Pyramid Butte, ND (Fig. 18F–G) is incomplete and lacks a terminal chamber. The preserved portion includes a loosely packed frass trail, which was deposited in a meniscate pattern (Fig. 18G). This frass pattern is not present on any Mexican Hat specimen, and the unique larval behavior necessary to produce the meniscate shape suggests that a different leaf-mining species created the mine. The mine on *Z. flabella* from YPM loc. 8403 (Fig. 18H) is very poorly preserved, but it shares some features associated with DT91 at Mexican Hat, in particular a continuous, medial frass trail composed of ellipsoidal-spheroidal pellets. The poor preservational quality prevents a full comparison of mine features, but it is probably related to the DT91 mines on *Z. flabella* at Mexican Hat.

Although members of the Platanaceae and Cercidiphyllaceae have been sampled extensively from both the local Cretaceous and Paleocene [6]–[8], [14], [39], we found no evidence that these plant families provided a refuge for Cretaceous leaf-mining insects (Table 6, 7). As described above, no mines found on Cretaceous specimens of either family were found on any confamilial or other Paleocene species.

**Table 6:** Comparison of leaf mine damage types on Cercidiphyllaceae leaves from the Cretaceous Hell Creek Formation, Williston Basin, North Dakota; local early Paleocene localities from the Fort Union Formation, Powder River and Williston Basins, of North Dakota and Montana; and Mexican Hat, Powder River Basin, Montana.

Mine DT	Hell Creek Fm.	Mexican Hat	Local Early Paleocene
DT35	X		
DT41		X	
DT42	X		
DT45	X		
DT65	X		
DT91		X	

**Table 7:** Comparison of mine damage types on Platanaceae leaves from the Cretaceous Hell Creek Formation, Williston Basin, North Dakota; local early Paleocene localities from the Fort Union Formation, Powder River and Williston Basins, of North Dakota and Montana; and Mexican Hat, Powder River Basin, Montana.

Mine DT	Hell Creek Fm.	Mexican Hat	Local Early Paleocene
DT35	X		
DT36		X	
DT41	X		
DT42	X		
DT43	X		
DT45	X		
DT65	X		
DT91		X	
DT104		X	
DT282		X	X

### Possible causes of leaf miner extinction

Two, intimately related scenarios for the extinction of specialized insects at the K-Pg boundary have been suggested: a direct extinction caused by catastrophic environmental conditions in the wake of the asteroid impact, and extinction caused by the loss of host plants [11]. Insect herbivores may track plant chemicals [87], [88] and switch hosts to plants with similar secondary chemistry, often including closely related plants. Although there were multiple Platanaceae and Cercidiphyllaceae species during the latest Cretaceous, and host-switching can occur on ecological time scales [89], [90], the sudden plant extinction and small surviving host-species pool may not have left enough time or options for host-switching to occur.

Because leaf-mining larvae are overwhelmingly host specialized [53], a lack of suitable hosts, even for a short time period, could cause extinction. Low light levels after the Chicxulub impact caused a decrease in photosynthesis for a period of months to years [91]–[93]. Angiosperms that crossed the K-Pg boundary may have survived in seed banks, germinating when conditions were suitable. Insect extinction during the extreme conditions directly after the impact, when plants were not growing, may well explain the low diversity of mines, even on boundary crossing plants.

### Why Mexican Hat? Possible causes of high damage diversity

What is different about Mexican Hat? The site captures an otherwise unknown “decoupled” [19] plant-insect ecosystem of high insect richness on depauperate Paleocene plants, but its flora and depositional environment were in no way unique for the region or time period. Its dominant plants were very widespread, and like Mexican Hat, the sediments of most regional Paleocene plant localities were deposited in floodplain and swamp environments [6]. In modern rainforests, plant genera with wide ranges can support insect faunas with low beta diversity [94], so low diversity Paleocene floras dominated by widespread species might be expected to support similar insect faunas across fossil localities. We did not observe any Paleocene sites with insect damage diversity comparable to Mexican Hat, even at Lebo Member localities deposited nearby and at an approximately similar time (Signal Butte, MT section [14], [30], [39], ca. 32 km from Mexican Hat). The high diversity of leaf-mining damage at Mexican Hat, and the lack of evidence linking the mines to local Cretaceous or Paleocene localities, instead suggest an influx of novel herbivores into the area, possibly within a very small spatial or temporal window.

Differences in preservational quality can contribute to variation in observed damage diversity among sites, but we do not find this to be explanatory. Leaf fossils at Mexican Hat are certainly well-preserved, and fourth or even fifth order venation is typically visible. There is a noticeable color contrast between the fossil leaf tissue and rock matrix that may increase leaf-mine visibility. Fossil preservation varied among localities in this study, ranging from poorly preserved (sandy sediments or faint fossil preservation) to preservation comparable to Mexican Hat. Most of the sites that are closest in age to Mexican Hat from the Williston Basin in North Dakota and the Powder River Basin in Montana have poorer preservation, but not nearly so poor, based on our collective experience, that leaf mines would falsely appear to be nearly absent. Despite differences in sediment size and preservation, Cretaceous leaf fossils in coarse-grained sandstones, such as at Mud Buttes and Battleship, preserved diverse leaf mine DTs on many host plants. The high preservational quality of the Mexican Hat flora probably somewhat affected observed insect damage diversity, but the presence of diverse leaf mine DTs even at the more poorly preserved Cretaceous sites suggests that causes other than taphonomic bias led to the patterns observed in this study.

Mexican Hat could represent a rare insect outbreak captured in the fossil record [95]. There are many causes of outbreaks, including changes in plant nutritional quality [96], changes in climate (increases in temperature, drought, etc.; [96], [97]), and reduced top-down control from predators or parasites [98]. However, modern insect outbreaks are typically associated with a sudden increase in population densities of a single insect herbivore species, whereas Mexican Hat documents high diversity at ordinal level, including agromyzid mines, rare hymenopteran mines on *P. raynoldsii*, and diverse Lepidopteran mines on seven host plants, along with a great variety of other damage. Also, fine-grained leaf-fossil deposits such as Mexican Hat are estimated to represent 500–1000 years of deposition [99], and insect-damaged leaves occur abundantly throughout the fossiliferous section, but insect outbreaks occur on much shorter time scales.

The increase in mining diversity more closely, and plausibly, resembles rapid increases in insect damage diversity associated with warming, suggesting that the Mexican Hat assemblage lived during a transient warming event that brought in a diverse immigrant fauna of minute flying insects that fed on the depauperate, less mobile plant assemblage and thus established the decoupled plant and insect diversity. Insect herbivory, including both damage diversity and intensity, is highest in the tropics [100], [101], implying a correlation between diversity and temperature. Increased insect herbivory in response to rising temperatures has been observed in modern agricultural and natural ecosystems [102], [103]. Most importantly here, several different warming events during the Paleocene-Eocene transition in Wyoming, including the Paleocene-Eocene Thermal Maximum (PETM), all led to significant increases in insect damage diversity, including that of mines and galls, on geologically short time scales [16], [18], [34]. High insect damage diversity has also been observed during the early Eocene climatic optimum in Patagonia [104] and in the middle Eocene of Germany [105], and thermophilic formiciine ants migrated across the Arctic into North America from Europe during hyperthermal conditions between the latest Paleocene and early middle Eocene [106]. Damage type diversity declined as temperatures decreased after the PETM in the western USA, suggesting that the prior increase in DT diversity was driven by climate change, and not a coincidental evolutionary radiation [16], [18]. Lower DT diversity has also been observed during cooler climates in the Oligocene of Ethiopia [107] and Germany [108].

Short hyperthermal events have been documented in early Paleocene marine sections, including during the early Danian (Dan-C2 event [109], [110], Top Chron 27 [111] or latest Danian event [112], and the Danian-Selandian transition event [113], [114]). The Dan-C2 event has been detected in marine carbonate δ^13^C and δ^18^O records in the Atlantic Ocean [109] and western Tethys [110], but not in the Pacific Ocean [115]. A terrestrial carbon isotope excursion, possibly concurrent with the Dan-C2 event, has also been observed at the Boltysh crater in Ukraine [116]. Pollen analysis there revealed an increase and dominance of thermophilic *Normapolles* pollen during the carbon isotope excursion, suggesting a shift to a warmer and drier climate [116]. The age resolution of the Mexican Hat strata is currently insufficient to correlate directly to these specific hyperthermal events, but more broadly, the existence of a number of short-lived marine hyperthermal events at this time suggests that an undocumented warming event during Mexican Hat deposition is a plausible scenario. More precise age constraints for Mexican Hat and the discovery and sampling of new localities of the same age are necessary to test the warming hypothesis. Paleogene hyperthermals were geologically brief, so it is very unlikely that any other local sites so far known were deposited during the same short time span. We hypothesize that if localities of precisely the same age as Mexican Hat are found, they will be associated with similar levels of damage.

We note that recovery of insect damage diversity after the K-Pg in the Western Interior USA finally began during late Paleocene warming, before the recovery of plant diversity [117], and this could be a general pattern given the potentially faster migration potential of some insect vs. plant taxa, especially winged insects with very small body sizes that are typical for the adult phases of leaf miners. Mexican Hat may represent a “failed” version of this pattern, wherein the proposed, short-lived increase in temperature led to an increase in local insect damage diversity via immigration. Current evidence suggests that impact effects on insect herbivores decreased with increasing distance from the Chicxulub crater [118]–[120], and thus pools of insect species would have existed elsewhere that could have sourced the influx observed at Mexican Hat. Insects can be rapidly transported long distances by wind as aerial plankton, and microlepidopterans, hymenopterans, and dipterans, including agromyzid flies, have been captured over oceanic waters 460 km from the nearest land [121]. In contrast, a short warming interval may not have left enough time for plant ranges to shift effectively. The low diversity, homogenous plant species diversity pool across a large geographic area during the early Paleocene [6]–[8], [14], [19] may have also impeded any similarly rapid increase in plant diversity. Modern ecological studies on much shorter time scales show a comparable pattern in response to temperature increase, with a disproportionate increase in insect biomass compared to plant biomass [122].

## Conclusions

Mexican Hat is the only known early Paleocene locality with high insect damage diversity on a typical, low-diversity flora. Our results suggest that the diversity of mining insects represented at Mexican Hat is greater than previously recognized, but there is no evidence linking any Cretaceous mines with those found at Mexican Hat. There is also no convincing evidence for the survival of any Cretaceous leaf miners over the K-Pg boundary regionally, even on the well-sampled, surviving plant families (Platanaceae and Cercidiphyllaceae). Cretaceous leaf mines are also much more morphologically diverse than those of the Paleocene. All these results suggest a severe regional extinction of leaf-mining insects rather than the survivorship of Cretaceous insect taxa. Our results strongly suggest that the high insect damage diversity on the low diversity Mexican Hat flora is linked to an influx of novel insect herbivores during the Paleocene, most plausibly caused by an undocumented, transient warming event that relocated tiny flying insects.

## Supporting Information

Table S1 **Rescored damage types on Mexican Hat census collection curated at USNM.**
(XLSX)

Table S2 **Mexican Hat damage types by host plant.**
(XLSX)

Table S3 **Leaf mines from local and regional Cretaceous and Paleocene localities.**
(XLSX)

Appendix S1 **Descriptions of new damage types.**
(DOCX)
