# Supplementary material for: Novel Insect Leaf-Mining after the End-Cretaceous Extinction and the Demise of Cretaceous Leaf Miners, Great Plains, USA
Source: PLoS One. 2014 Jul 24;9(7):e103542. doi: 10.1371/journal.pone.0103542 (PMC4110055; doi:10.1371/journal.pone.0103542)
Supplement: Appendix S1 — Descriptions of new damage types. (DOCX) [file pone.0103542.s004.docx]

**Appendix S1. Descriptions of new damage types.**

**DT281**

Short Description: Pattern of puncture marks clustered in an ellipse or circle

Long Description: Circular, ellipsoidal, and comma shaped puncture marks clustered in an ellipse or circle. Clusters are usually associated with a primary or secondary vein and puncture marks tend to increase in size towards the center of the cluster with the largest marks positioned adjacent to the major vein. Individual marks range in diameter from 0.19 to 0.43 mm. The marks are mostly positioned around primary or secondary veins, but they are also found on interveinal areas.

Repository: Smithsonian Institution, National Museum of Natural History, Department of Paleobiology, Washington, DC, USA (USNM)

Reference specimen: USNM 560163 (MH362)

Fort Union Formation, Locality USNM 42090 (Mexican Hat), Powder River Basin, Montana, USA

Age: early Paleocene (Danian)

Host: Lauraceae sp. 2 at Mexican Hat

Figured: 12I-K

**DT282**

Short Description: Epidermal mine; serpentine; no width expansion; margins delimited by dark frass or reaction tissue

Long Description: A serpentine mine, likely through epidermal tissue, with no width increase. Dark frass or reaction tissue occurs along the margins and takes up 25-75% of the overall width of the mine. The center of the mine path is free of frass. Leaf venation does not affect the course of the mine.

Repository: Yale University, Peabody Museum of Natural History, Division of Paleobotany, New Haven, CT, USA (YPM)

Reference specimen: YPM 9796

Fort Union Formation, Locality YPM 86107, Williston Basin, North Dakota, USA (also found in Fort Union Formation, Locality USNM 42090 (Mexican Hat), Powder River Basin, Montana, USA)

Age: early Paleocene (Danian)

Hosts: *“Populus” nebrascensis* (unknown affinity) at Pyramid Butte, ND, *Platanus raynoldsii* (Platanaceae) at Mexican Hat, MT, *Juglandiphyllites glabra* (Juglandaceae) at Mexican Hat, MT

Figured: 3E, 4K, 18C-D
